# The burden of chronic respiratory disease and attributable risk factors in North Africa and Middle East: findings from global burden of disease study (GBD) 2019

**DOI:** 10.1186/s12931-022-02187-3

**Published:** 2022-09-29

**Authors:** Aida Fallahzadeh, Yeganeh Sharifnejad Tehrani, Ali Sheikhy, Seyyed-Hadi Ghamari, Esmaeil Mohammadi, Sahar Saeedi Moghaddam, Zahra Esfahani, Maryam Nasserinejad, Parnian Shobeiri, Mohammad-Mahdi Rashidi, Nazila Rezaei, Mahsa Heidari-Foroozan, Negar Rezaei, Bagher Larijani, Farshad Farzadfar

**Affiliations:** 1 Non-Communicable Diseases Research Center, Endocrinology and Metabolism Population Sciences Institute, Tehran University of Medical Sciences, Tehran, Iran; 2 Department of Biostatistics, University of Social Welfare and Rehabilitation Sciences, Tehran, Iran; 3 Department of Biostatistics, Faculty of Paramedical Sciences, Shahid Beheshti University of Medical Sciences, Tehran, Iran; 4 Endocrinology and Metabolism Research Center, Endocrinology and Metabolism Clinical Sciences Institute, Tehran University of Medical Sciences, Tehran, Iran

**Keywords:** Burden, Chronic respiratory disease, DALYs, GBD, North Africa and Middle East

## Abstract

**Background:**

North Africa and Middle East (NAME) has an increasing burden of chronic respiratory diseases (CRDs); however, a systematic understanding of the distribution and trends is not available. We aimed to report the trends of CRDs and attributable risk factors in this region between 1990 and 2019.

**Methods:**

Using data from the Global Burden of Diseases Study (GBD) 2019, cause specific mortality served as the basis for estimating incidence and disability-adjusted life years (DALYs). The burden attributable to risk factors was calculated by a comparative risk assessment and contribution of population ageing and growth was determined by decomposition analysis.

**Results:**

The number of deaths due to CRD in 2019 were 128,513 (110,781 to 114,351). In 2019, the age-standardized incidence rate (ASIR) of CRDs was 1052.8 (924.3 to 1209.4) per 100,000 population and had a 10.3% increase and the age-standardized death rate (ASDR) was 36.1 (30.9 to 40.3) with a 32.9% decrease compared to 1990. In 2019, United Arab Emirates had the highest ASIR (1412.7 [1237.3 to 1622.2]) and Afghanistan had the highest ASDR (67.8 [52.0 to 81.3]). CRDs were responsible for 2.91% of total DALYs in 2019 (1.69% due to chronic obstructive pulmonary disease [COPD] and 1.02% due to asthma). With regard to the components of DALYs, the age-standardized rate of years of life lost (YLL) had a − 39.0% (− 47.1 to − 30.3) decrease; while the age-standardized rate of years lived with disability (YLD) had a 13.4% (9.5 to 17.7) increase. Of total ASDRs of CRDs, 31.6% were attributable to smoking and 14.4% to ambient particulate matter pollution.

**Conclusion:**

CRDs remain a leading cause of death and disability in NAME, with growth in absolute numbers. COPD and asthma were the most common CRDs and smoking was the leading risk factor especially in men. More attention is needed in order to reduce CRDs’ burden through appropriate interventions and policies.

**Supplementary Information:**

The online version contains supplementary material available at 10.1186/s12931-022-02187-3.

## Background

Chronic respiratory diseases (CRDs) are among the leading non-communicable diseases (NCDs) and important contributors to the rising burden of NCDs in North Africa and Middle East (NAME) [1]. Compared with other NCDs, CRDs were neglected for many years [2, 3]. In this region, CRDs reached from 12th cause of all ages death in 1990 to 6th cause of death in 2019 and were responsible for 2.91% of total disability-adjusted life years (DALYs) and 4.14% of total deaths in 2019 [4].

Several common modifiable risk factors have been identified for CRDs, such as tobacco smoking, second-hand smoking, indoor and outdoor pollutions, allergens, and occupational agents [5]. Moreover, there is a relationship between CRDs and risk of pulmonary neoplasms and tobacco smoking is a common risk factor for CRDs and lung cancer [6]. Thus, identifying the trends and planning for prevention of these potential risk factors will have a significant impact on morbidity and mortality. According to world health organization (WHO) global reports, while tobacco use, as the main risk factor for CRDs, is decreasing worldwide, the prevalence of tobacco smoking in the NAME region is expected to increase between 2010 and 2025 [7].

Previous studies have estimated the prevalence and burden of CRDs and attributed risk factors at the global level [8, 9] and showed that the prevalence of CRDs in NAME increased between 1990 and 2017 and was estimated to be 7.7% in 2017 [9]. However, comprehensive data regarding the epidemiology and burden of CRDs in the NAME region are lacking. Thus, in this study we provided a systematic report of CRDs in the NAME region including incidence, prevalence, mortality and DALYs and compared them by sex, age groups, countries and socio-demographic index (SDI) based on the Global Burden of Diseases, Injuries, and Risk Factors Study (GBD) 2019 [10], which is an essential step to apply policies to reduce the burden of CRDs in this region.

## Material and methods

The GBD 2019 study, conducted by the Institute for Health Metrics and Evaluation (IHME), involved 21 geographical regions composed of 204 countries and territories from 1990 to 2019 [10]. The original input data for CRDs, as well as the modelling strategy for death, were described in detail previously [10]. Herein, we focused on the methods and statistical analyses of estimation the CRDs burden from the GBD 2019 study. The NAME region consists of 21 countries including Afghanistan, Algeria, Bahrain, Egypt, Iran, Iraq, Jordan, Kuwait, Lebanon, Libya, Morocco, Oman, Palestine, Qatar, Saudi Arabia, Sudan, Syrian Arab Republic, Tunisia, Turkey, United Arab Emirates, and Yemen.

As per GBD Data Dictionary, CRDs include the following five categories: asthma, chronic obstructive pulmonary disease (COPD), interstitial lung disease (ILD) and pulmonary sarcoidosis, pneumoconiosis (including silicosis, asbestosis, coal-worker pneumoconiosis, and other pneumoconiosis), and other CRDs. The International Classification of Diseases (ICD)-10 codes were used for mapping the GBD cause list (Additional file 1).

The prevalence of CRDs was estimated by using DisMod-MR version 2.1, a Bayesian regression analytical tool used in the GBD study [10, 11]. All-cause mortality rates were derived from vital registration systems, censuses, and surveys, and were analyzed with demographic methods to correct for incompleteness. Cause of Death Ensemble model (CODEm) [12] tool was used to generate CRDs mortality estimates. CoDCorrect was used to ensure that the independently modeled single-cause mortality estimates match the separately modeled all-cause mortality [10]. Mortality-to-incidence ratios (MIR) were applied to the final CRD’s mortality measures to estimate the incidence. To estimate YLLs, each death caused by CRD was multiplied by the standard life expectancy at that age. YLDs were calculated by multiplying prevalence of each sequela by the sequela specific disability weight. A brief measure of cause-burden based on both non-fatal health losses and premature deaths was reported as DALYs, which were the addition of the YLLs and YLDs.

Risk factors’ burdens were estimated consistent with general framework established for comparative risk assessment (CRA) [10, 11, 13]. CRA consists of risk-outcome pair inclusion, relative risk estimation, exposure level and distributions estimation, counterfactual level of exposure determination, and population attributed fractions and burden computation [11].

Estimates were composed of 12 behavioral, environmental/ occupational, and metabolic risk factors for CRDs (Ambient ozone pollution, Ambient particulate matter pollution, high body-mass index [BMI], High temperature, Household air pollution from solid fuels, Low temperature, Occupational asthmagens, Occupational exposure to asbestosis, Occupational exposure to silica, Occupational particulate matters, gases, and fumes, Second hand smoke, and Smoking) [11].

The Socio-demographic Index (SDI) is a composite indicator of development status which is strongly correlated with health outcomes. It is the geometric mean of 0 to 1 indices of total fertility rate under the age of 25, mean education for those ages 15 and older and lag distributed income per capita [14]. Each country in the region was grouped based on SDI into 5 SDI quintiles, including low, low-middle, middle, high-middle and high (Additional file 2). Decomposition analysis was conducted to analyze the contribution of population aging, population growth, and changes in the age-specific incidence rates on the absolute change of CRD incidence [15].

### Statistical analysis

Data will be described in terms of absolute numbers and age-standardized rates per 100,000 population following 95% uncertainty interval (UI), 25th and 95th values of the ordered draws. All the statistical analyses, plots, and numbers created in this study were performed by R for windows v 4.0.3 [16].

## Results

### Incidence and prevalence

**Fig. 1:**
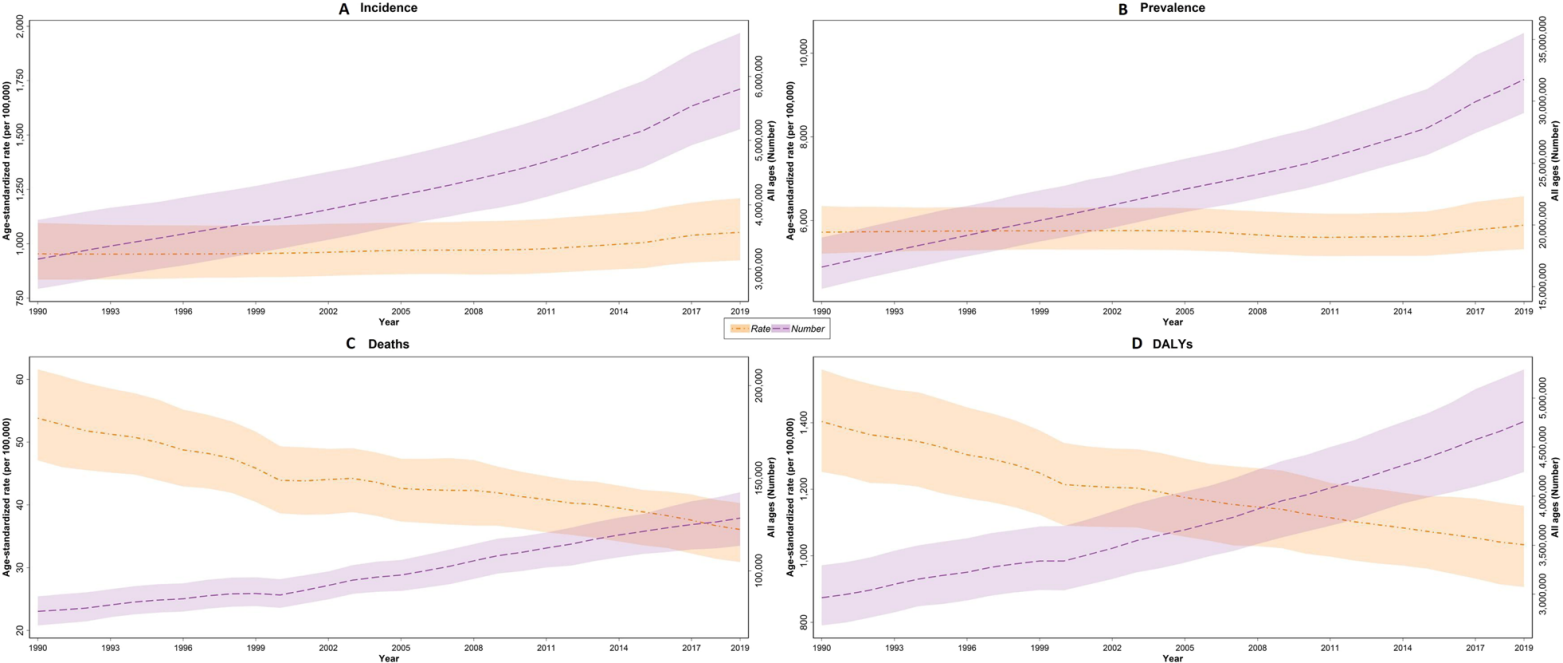
The time trend of all ages number and age-standardized rate of CRDs. **A** Incidence, **B** prevalence, **C** deaths, and **D** DALYs

The CRDs incident cases increased from 3,154,020 (95% UI 2,622,223 to 3,814,340) in 1990 to 5,803,364 (5,049,564 to 6,767,369) in 2019, revealing a 1.8-fold increase; moreover, the age-standardized incidence rate (ASIR) of CRDs increased from 954.2 (835.8 to 1095.5) in 1990 to 1052.8 (924.3 to 1209.4) per 100,000 population in 2019 (Table 1 and Fig. 1A). Between 1990 and 2019, ASIR increased in all countries, except for 4 countries (Bahrain, Iran, Iraq, and Yemen) with a decrease in ASIR. United Arab Emirates had the highest ASIR in 2019 (1412.7 [1237.3 to 1622.2] per 100,000 population) (Additional file 3). Asthma had the highest ASIR (589.8 [473.1 to 728.9] per 100,000 population) and pneumoconiosis had the lowest ASIR (0.9 [0.7 to 1.1] per 100,000 population) in 2019. ASIR of pneumoconiosis decreased in men (− 32.8% [− 42.2 to − 21.5]) and increased in women (70.3% [60.4 to 81.9]) (Table 2, Fig. 1A).

**Table 1:** Chronic respiratory disease burden by measure in 2019, with percentage change by sex

Measure	Sex	Incidence		Prevalence		Deaths		DALYs		YLLs		YLDs	
		Number (95% UI)	Rate (95% UI)	Number (95% UI)	Rate (95% UI)	Number (95% UI)	Rate (95% UI)	Number (95% UI)	Rate (95% UI)	Number (95% UI)	Rate (95% UI)	Number (95% UI)	Rate (95% UI)
Burden	Female	2,892,958 (2,532,131 to 3,337,602)	1082.1 (955.9 to 1234.7)	15,508,605 (13,874,856 to 17,541,972)	5887.6 (5331.7 to 6538.2)	51,001 (41,322 to 59,279)	29.1 (23.3 to 33.6)	2,061,757 (1,756,716 to 2,352,145)	899.7 (772.2 to 1017.4)	1,089,221 (901,390 to 1,292,329)	519.2 (430.9 to 610.8)	972,536 (745,075 to 1,235,904)	380.5 (294.1 to 474.8)
	Male	2,910,406 (2,493,934 to 3,409,244)	1026.5 (895.4 to 1187.3)	16,267,592 (14,472,613 to 18,550,695)	5909.4 (5343.8 to 6630.6)	77,512 (66,773 to 88,312)	43.0 (37.0 to 48.6)	2,697,848 (2,337,411 to 3,055,791)	1165.2 (1023.3 to 1307.5)	1,681,314 (1,423,688 to 1,923,462)	779.0 (667.0 to 890.1)	1,016,534 (778,068 to 1,291,282)	386.1 (297.5 to 479.3)
	Both	5,803,364 (5,049,564 to 6,767,369)	1052.8 (924.3 to 1209.4)	31,776,197 (28,349,827–36,022,813)	5891.2 (5321.0 to 6584.3)	128,513 (110,781 to 144,351)	36.1 (30.9 to 40.3)	4,759,606 (4,142,498 to 5,361,709)	1033.4 (906.7 to 1149.3)	2,770,536 (2,390,411 to 3,148,496)	650.7 (563.8 to 733.1)	1,989,070 (1,525,254 to 2,518,856)	382.8 (298.4 to 474.7)
% Change (1990–2019)	Female	83.4 (71.5 to 96.5)	8.8 (5.3 to 12.3)	86.8 (78.2 to 95.3)	− 0.4 (− 3.6 to 2.8)	56.2 (27.4 to 99.7)	− 34.3 (− 47.5 to − 15.8)	54.5 (37.3 to 83.5)	− 27.2 (− 36.3 to − 14.5)	24 (3.1 to 63.7)	− 41.5 (− 52.2 to − 25.2)	113.3 (101.4 to 127.2)	9.3 (5.4 to 13.3)
	Male	84.6 (72.7 to 98.7)	12.1 (8.6 to 15.7)	96.3 (87.6 to 105.5)	6.6 (3.6 to 9.8)	70.3 (41.9 to 102.1)	− 32.6 (− 44.6 to − 20.3)	65.5 (47.5 to 87.2)	− 25.9 (− 35.4 to − 15.7)	42.1 (19.9 to 70.3)	− 37.4 (− 47.9 to − 25.5)	127.5 (112.0 to 145.8)	17.8 (13.3 to 23.0)
	Both	84.0 (72.4 to 97.0)	10.3 (7.1 to 13.5)	91.6 (83.1 to 100.0)	2.9 (0.0 to 5.9)	64.4 (42.5 to 88.4)	− 32.9 (− 42.0 to − 23.4)	60.6 (47.1 to 75.9)	− 26.4 (− 33.4 to − 19.1)	34.4 (17.1 to 54.6)	− 39.0 (− 47.1 to − 30.3)	120.3 (106.7 to 135.8)	13.4 (9.5 to 17.7)
Attributed burden to all risk factors	Female	n/a	n/a	n/a	n/a	25,661 (20,192 to 30,506)	14.8 (11.3 to 17.5)	859,987 (711,145 to 1,017,698)	395.2 (327.4 to 465.7)	516,635 (409,747 to 620,767)	253.5 (201.9 to 302.8)	343,352 (264,282 to 430,448)	141.7 (110.0 to 174.3)
	Male	n/a	n/a	n/a	n/a	57,039 (47,854 to 65,008)	32.0 (27.0 to 36.0)	1,707,278 (1,466,720 to 1,924,884)	780.5 (676.9 to 874.6)	1,179,486 (984,420 to 1,359,299)	561.1 (469.9 to 643.7)	527,792 (413,000 to 644,058)	219.4 (173.4 to 262.9)
	Both	n/a	n/a	n/a	n/a	82,700 (70,521 to 93,662)	23.3 (19.9 to 26.4)	2,567,265 (2,221,981 to 2,890,442)	590.1 (513.9 to 662.4)	1,696,120 (1,431,688 to 1,949,413)	409.1 (347.4 to 465.5)	871,144 (684,153 to 1,066,190)	181.0 (143.1 to 216.8)
% Change (1990–2019)	Female	n/a	n/a	n/a	n/a	71.5 (37.3 to 131.2)	− 30.8 (− 45.5 to − 7.3)	82.4 (57.1 to 122.6)	− 25.4 (− 36.0 to − 7.9)	52.6 (23.0 to 106.5)	− -36.5 (− 49.0 to − 14.3)	158.4 (144.9 to 172.7)	8.6 (3.1 to 14.7)
	Male	n/a	n/a	n/a	n/a	86.6 (52.5 to 123.3)	− 28.3 (− 41.1 to − 14.1)	95.7 (67.6 to 125.9)	− 22.3 (− 34.0 to − 10.0)	69.7 (36.6 to 104.4)	− 32.2 (− 45.0 to − 18.4)	198.0 (187.1 to 210.6)	23.7 (19.5 to 28.7)
	Both	n/a	n/a	n/a	n/a	81.6 (57.3 to 109.7)	− 28.3 (− 38.4 to − 17.7)	91.1 (71.5 to 113.6)	− 23.0 (− 31.0 to − 13.8)	64.1 (41.7 to 91.2)	− 33.2 (− 42.4 to − 22.5)	181.0 (170.4 to 192.5)	17.7 (13.5 to 22.4)

Data in parentheses are 95% uncertainty intervals (95% UIs)

*DALYs* disability-adjusted life years, *YLLs* years of life lost, *YLDs* years lived with disability

**Table 2:** Age-standardized burden of chronic respiratory diseases in 2019, with percentage change by sex

Measure	Sex	COPD		Pneumoconiosis		Asthma		ILD and sarcoidosis		Other CRDs	
		2019 (95% UI)	% Change (1990–2019)	2019 (95% UI)	% Change (1990–2019)	2019 (95% UI)	% Change (1990–2019)	2019 (95% UI)	% Change (1990–2019)	2019 (95% UI)	% Change (1990–2019)
ASIR	Female	121.3 (113.9 to 128.9)	26.8 (23.8 to 29.9)	1.7 (1.3 to 2.1)	70.3 (60.4 to 81.9)	617.2 (506.8 to 752.3)	− 4.2 (− 9.2 to 0.2)	342.0 (281.3 to 410.8)	34.8 (30.2 to 39.2)	n/a	n/a
	Male	179.3 (171.1 to 187.9)	39.7 (36.3 to 43.3)	0.2 (0.2 to 0.2)	− 32.8 (− 42.2 to − 21.5)	564.3 (442.2 to 711.7)	− 3.5 (− 8.7 to 1.4)	282.7 (232.7 to 333.8)	39.8 (34.8 to 45.2)	n/a	n/a
ASPR	Female	1981.2 (1876.6 to 2093.3)	23.4 (20.8 to 26.1)	4.1 (3.2 to 5.1)	82.9 (69.7 to 101.1)	4150.0 (3605.6 to 4833.4)	− 8.3 (− 12.7 to − 4.0)	49.5 (42.4 to 57.0)	39.5 (35.1 to 43.9)	n/a	n/a
	Male	2681.6 (2575.5 to 2793.8)	36.0 (33.2 to 39.3)	1.1 (1.0 to 1.4)	− 32.8 (− 42.2 to − 21.5)	3508.7 (2933.2 to 4244.6)	− 7.1 (− 12.0 to − 1.6)	39.9 (34.3 to 46.1)	45.1 (39.9 to 50.3)	n/a	n/a
ASDR	Female	19.5 (14.7 to 22.6)	− 18.2 (− 35.4 to 28.7)	0 (0 to 0)	56.9 (14.7 to 119.9)	8.2 (6.5 to 10.1)	− 57.2 (− 70.7 to − 45.3)	1.2 (0.8 to 2.2)	2.9 (− 20.4 to 54.1)	0.3 (0.2 to 0.4)	2.8 (− 39.7 to 66.8)
	Male	32.6 (27.5 to 37.1)	− 18.9 (− 34.3 to 0.3)	0.1 (0.1 to 0.1)	− 27.5 (− 49.0 to 34.2)	8.6 (6.9 to 10.9)	− 60.6 (− 68.1 to − 46.9)	1.3 (0.9 to 2.2)	6.0 (− 20.5 to 92.3)	0.4 (0.2 to 0.5)	18.0 (− 33.2 to 111.3)
Age-standardized DALYs	Female	509.1 (430.2 to 569.2)	− 12.3 (− 25.0 to 23.4)	1.4 (1.1 to 1.8)	70.3 (43.2 to 108.6)	331.6 (263 to 418.6)	− 45.4 (− 57.1 to − 34.6)	29.6 (21.2 to 51.2)	9.1 (− 13.5 to 47.0)	28 (23.5 to 32.8)	42.6 (− 2.1 to 81.6)
	Male	786.2 (686.7 to 883)	− 12.0 (− 25.4 to 3.1)	2.6 (2.1 to 3.1)	− 35.3 (− 54.1 to 9.6)	317.5 (248.8 to 394.4)	− 49.5 (− 57.4 to − 37.6)	30.5 (23.4 to 46.7)	10.4 (− 17.9 to 74.8)	28.4 (23.1 to 33.5)	42.5 (− 2.3 to 89.1)
Age-standardized YLLs	Female	313.4 (239.3 to 366.1)	− 25.5 (− 39.5 to 24.3)	0.8 (0.6 to 1.0)	64.5 (23.5 to 132.6)	171.5 (135.6 to 216.1)	− 60.4 (− 71.4 to − 49.8)	24.5 (16.6 to 46.2)	4.9 (− 18.8 to 51.2)	9.1 (6.1 to 12.7)	− 11.7 (− 49.9 to 49.5)
	Male	558.3 (462.5 to 644.6)	− 22.9 (− 38.0 to − 6.0)	2.4 (1.9 to 2.9)	− 37.5 (− 56.4 to 8.9)	179.7 (142.1 to 230.1)	− 62.6 (− 69 to − 50.4)	26.3 (19.4 to 42.3)	6.9 (− 22.0 to 80.8)	12.3 (8.1 to 16.0)	1.9 (− 41.4 to 71.8)
Age-standardized YLDs	Female	195.7 (160.8 to 222.7)	22.3 (19.0 to 25.7)	0.6 (0.4 to 0.9)	78.0 (63.2 to 96.3)	160.1 (104.3 to 236.1)	− 8.2 (− 12.7 to − 3.5)	5.1 (3.4 to 7.5)	35.4 (26.0 to 45.6)	18.9 (15.2 to 22.2)	102.8 (92.2 to 113.4)
	Male	227.8 (183.1 to 266.7)	34.8 (31.1 to 38.4)	0.2 (0.1 to 0.3)	21.8 (7.9 to 36.4)	137.8 (87.7 to 205.1)	− 6.8 (− 11.9 to − 1.1)	4.2 (2.8 to 6.2)	39.3 (30.2 to 50.3)	16.1 (12.6 to 19.7)	105.2 (94.8 to 115.9)

Data in parentheses are 95% uncertainty intervals (95% UIs)

*ASIR* age-standardized incidence rate, *ASPR* age-standardized prevalence rate, *ASDR* age-standardized death rate, *DALYs* disability-adjusted life years, *YLLs* years of life lost, *YLDs* years lived with disability, *COPD* chronic obstructive pulmonary disease, *ILD* interstitial lung disease, *CRD* chronic respiratory disease

The absolute number of CRDs prevalent cases and age-standardized prevalence rate (ASPR) in NAME increased during 1990 and 2019 (Fig. 1B and Table 1). Among all 21 countries in 2019, United Arab Emirates had the highest ASPR (9499.6 [8528.5 to 10,588.0], per 100,000 population) (Additional file 3, Fig. 2). Prevalent cases and ASPR of all CRD’s subtypes increased between 1990 and 2019, except for ASPR of asthma which decreased (− 7.9% [− 12.4 to − 3.2]) in both sexes (Table 2, Fig. 1B).

**Fig. 2:**
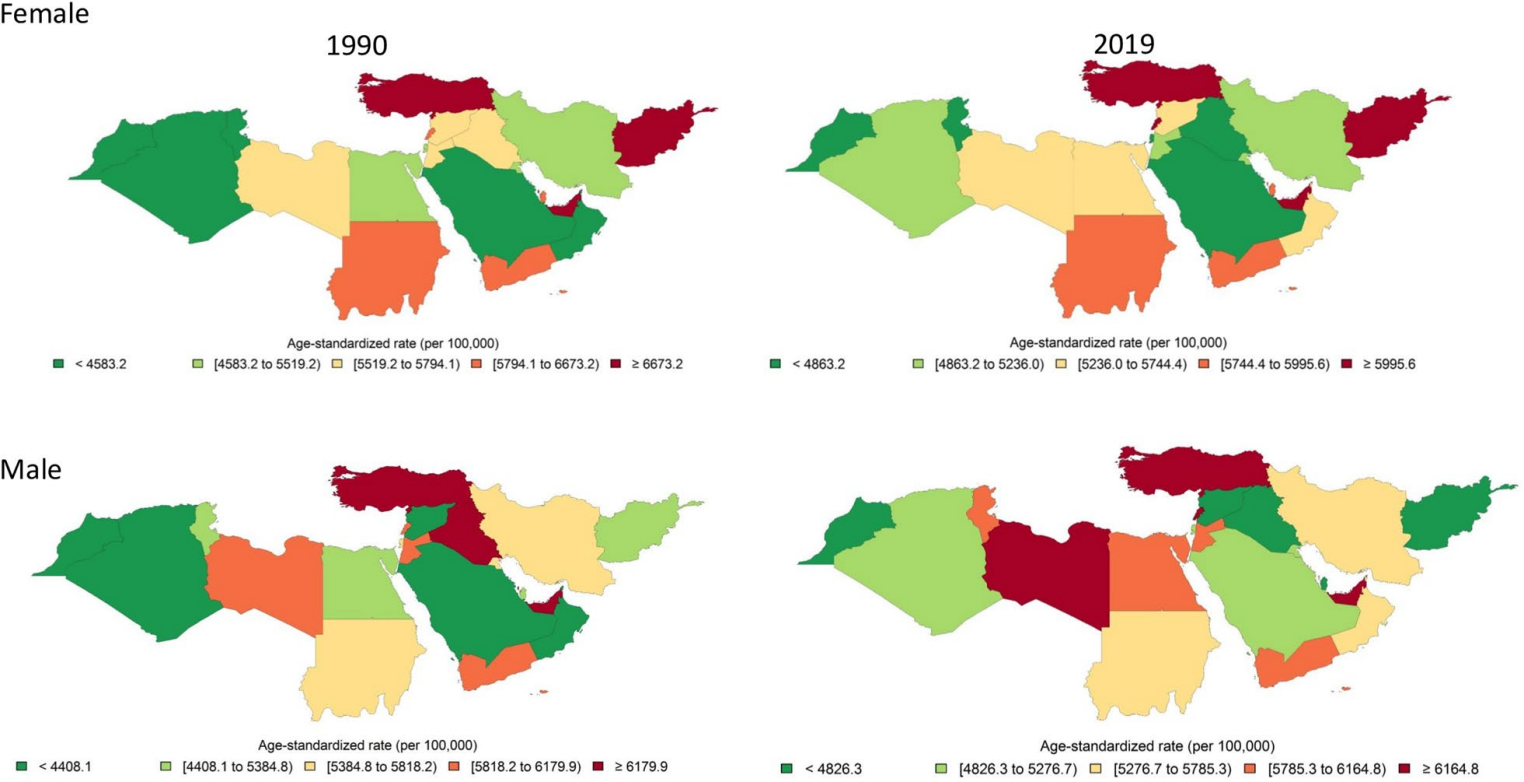
Age-standardized prevalence rate of CRDs in North Africa and Middle East countries in female and males in 1990 and 2019

**Fig. 3:**
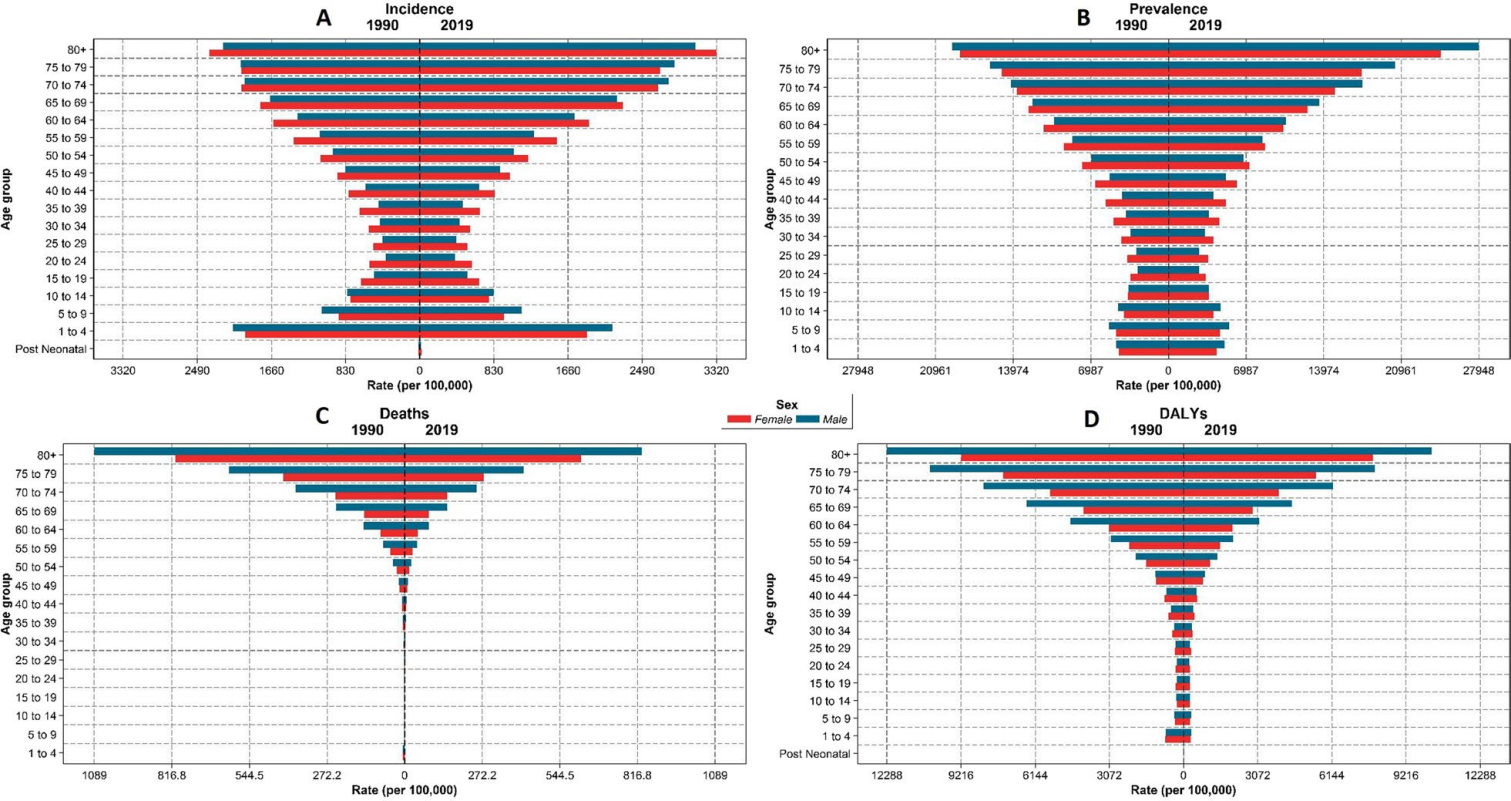
The rate of CRDs. **A** Incidence, **B** prevalence, **C** deaths, and **D** DALYs categorized by age groups and sex in 1990 and 2019

**Fig. 4:**
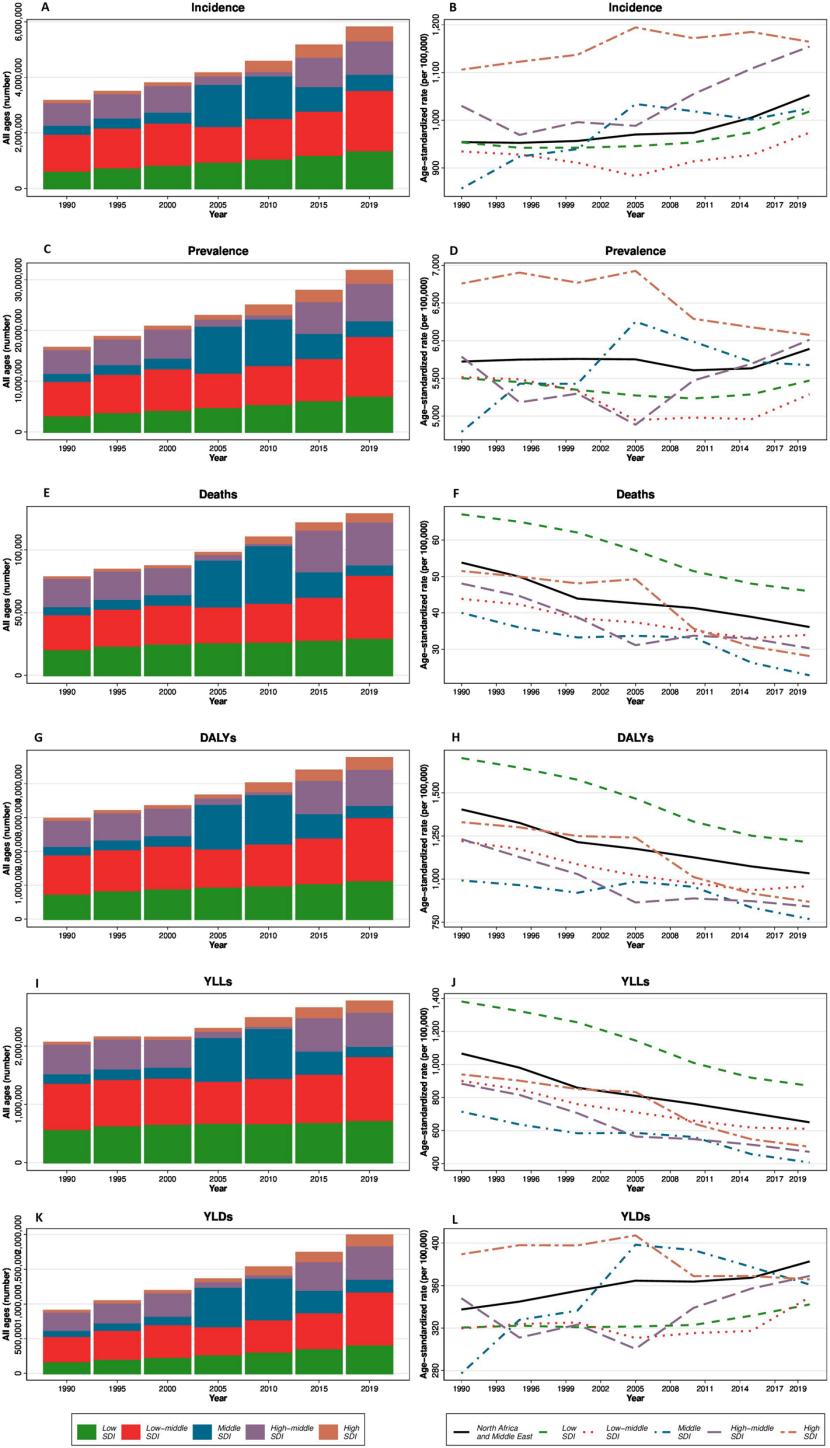
The time trend of all ages number and age-standardized rate of CRDs. **A**, **B** Incidence, **C**, **D** prevalence, **E**, **F** deaths, **G**, **H** DALYs, **I**, **J** YLLs, and **K**, **L** YLDs categorized by 5 SDI quintiles

With regard to the age groups, in 2019, the highest rate of incidence and prevalence was seen in 80 plus age group (incidence rate: 3204.0 [2794.4 to 3688.0], prevalence rate: 26,222.3 [24506.1 to 28,182.2] per 100,000 population). Among under 14 years population, the highest incidence rate was in 1–4 years age group (2016.3 [1256.6 to 3067.6] per 100,000 population) (Fig. 3). ASIR and number of incident cases increased in all SDI quantiles between 1990 and 2019 (Fig. 4).

Decomposition analysis of new cases in NAME showed an overall 84.0% increase from 1990 to 2019, of which 76.4% and 13.1% were attributed to population growth and incidence rate change, respectively; while, changes in age structure were responsible for − 5.6% decrease in this change (Additional file 4).

### Mortality

The number of CRDs deaths in NAME increased from 78,174 (68,863 to 87,436) in 1990 to 128,513 (110,781 to 114,351); while the age-standardized death rate (ASDR) of CRDs decreased from 53.8 (47.1 to 61.6) in 1990 to 36.1 (30.9 to 40.3) in 2019 per 100,000 population (Table 1, Fig. 1C). Among all countries in 2019, the highest ASDR was in Afghanistan (67.8 [52.0 to 81.3] per 100,000 population) and ASDR decreased in all countries (Additional file 3).

In 2019, COPD had the highest ASDR (26.1 [22.2 to 29.5] per 100,000 population). ASDR of COPD and asthma decreased in both sexes (− 18.0% [− 30.9 to − 2.8] and − 59.0% [− 68.2 to − 49.3]) while, ASDR of pneumoconiosis increased in females (56.9% [14.7 to 119.9]) (Table 2, Fig. 1C).

The highest rate of death was seen in 80 plus age group (723.7 [598.1 to 813.0] per 100,000 population) and the lowest was in age group of 10–14 years (0.6 [0.5 to 0.7] per 100,000 population) (Fig. 3). The number of CRDs death increased in all SDI quantiles during the study period, while the ASDR decreased in all SDIs (Fig. 4).

### DALYs, years of life lost (YLLs), years lived with disability (YLDs)

In NAME, CRDs were responsible for 2.91% of total DALYs in 2019, including 1.69% due to COPD, 1.02% due to asthma, and 0.1% due to other CRDs. The number of CRD’s DALYs increased from 2,964,169 (2,615,504 to 3,338,882) in 1990 to 4,759,606 (4,142,498 to 5,361,709) in 2019; while, the age-standardized DALYs rate decreased from 1403.7 (1252.6 to 1560.5) in 1990 to 1033.4 (906.7 to 1149.3) in 2019 per 100,000 population. With regard to the components of DALYs, the age-standardized rate of YLL had a − 39.0% (− 47.1 to − 30.3) decrease; while the age-standardized rate of YLD had a 13.4% (9.5 to 17.7) increase (Table 1 and Fig. 1D). Afghanistan had the highest age-standardized rate of DALYs in 2019 (1754.0 [1422.5 to 2077.1] per 100,000 population) (Additional file 3). The age-standardized DALYs rates of COPD and asthma decreased in both sexes (− 11.8% [− 21.1 to − 0.9] and − 47.6% [− 56.0 to − 39.1], respectively) and the age-standardized DALYs of pneumoconiosis increased in females (70.3% [43.2 to 108.6]) (Table 2, Fig. 1D). During 1990 and 2019, rate of DALYs due to CRDs was the highest in 80 plus age group (9045.1 [7870.5 to 9992.2], per 100,000 population) compared to other groups. Also, DALYs rate of CRDs had a decrease in all age groups during this period (Fig. 3). During the study period, age-standardized DALYs and YLL rates decreased in all SDI quantiles; while, the age-standardized rate of YLD showed an increasing pattern in all SDIs except in high-SDI countries (Fig. 4).

### Burden attributed to risk factors

In 2019, the CRDs ASDR and age-standardized DALYs rate attributable to all risk factors in NAME were 23.3 (19.9 to 26.4) and 590.1 (513.9 to 662.4) per 100,000 population and had − 28.3% (− 38.4 to − 17.7) and − 23.0% (− 31.0 to − 13.8) decline during the follow up period, respectively (Table 1). Of total ASDRs of CRDs, 31.6% (28.7 to 34.4) were attributable to smoking, 14.4% (11.2 to 18.2) to ambient particulate matter pollution, 8.7% (5.7 to 12.2) to high BMI, 7.5% (6.1 to 8.8) to occupational particulate matter, gases, and fumes, 6.3% (3.3 to 9.6) to secondhand smoke, 5.3% (4.0 to 6.5) to low temperature, and 4.8% (2.3 to 7.3) to ambient ozone pollution.

**Fig. 5:**
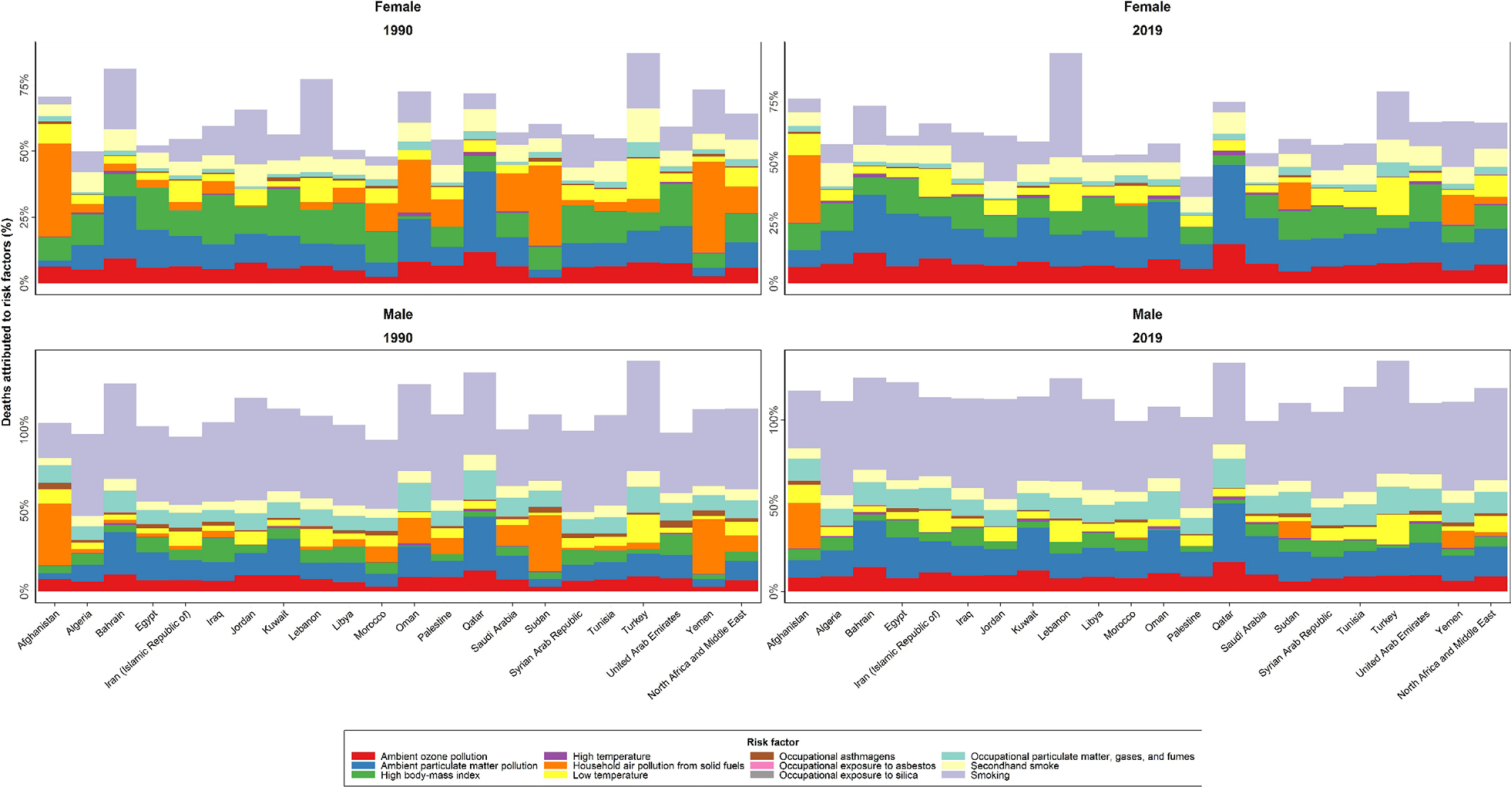
The age-standardized death rates of CRDs attributed to risk factors in North Africa and Middle East countries and region by sex in 1990 and 2019

Between 1990 and 2019, the ASDR of attributable percentage to occupational exposure to asbestoses (62.3% [10.2 to 176.6]), ambient particulate matter pollution (50.7% [33.2 to 77.0]), and ambient ozone pollution (28.8% [19.4 to 51.2]) increased, while the ASDR of attributed percentage to household air pollution from solid fuels (− 76.4% [− 81.9 to − 71.0]), and occupational asthmagens (− 40.6% [− 50.4 to − 25.0]) decreased in all countries (Fig. 5).

The ASDR of COPD in males were mainly attributed to smoking followed by ambient particular matter pollution and occupational particulate matter, gases, and fumes and in females to ambient particular matter pollution followed by smoking and low temperature, with slight changes between 1990 and 2019. The ASDR of asthma were mainly attributed to high BMI, smoking and occupational asthmagens in both sexes and the ASDR of pneumoconiosis were attributed to occupational exposure to silica and occupational particulate matter (Additional file 5).

## Discussion

In NAME, the ASIR of CRDs increased by 10.3% and the age-standardized DALYs rate of CRDs declined significantly by 26.4% between 1990 and 2019. This reduction was mainly caused by the reduction in ASDR and age-standardized YLL rate, while the age-standardized rate of YLD increased significantly. These findings may emphasize greater improvements in case fatality rather than changes in incidence and prevalence in the region. ASIR and ASPR were the highest in high-SDI, while ASDR and age-standardized DALYs were the highest in low-SDI countries, findings unchanged since first estimated in 1990. Asthma was the most prevalent CRD, affecting an estimated 22 million people in 2019 and COPD was half as common, with 11 million people affected. Although smoking is the leading risk factor for DALYs and deaths related to CRDs for men, other risk factors such as household and ambient particulate matter pollution and high BMI have considerable effects on women.

According to the increasing incidence and prevalence of CRDs and high rates of mortality in countries such as Afghanistan, increased attention and resources should be allocated to this category of diseases. According to the 2019 world population ageing report, the number of persons aged 80 and over significantly increased in the region between 1990 and 2019 and is estimated to show a high increase from 2019 to 2050 [17]. Moreover, in our study the highest incidence, prevalence, deaths and DALYs due to CRDs were concentrated in people aged 80 and over. Thus, the incidence and prevalence of CRDs are likely to continue increasing in the future. Moreover, COVID-19 pandemic which has happened during last months of 2019, could also affect the incidence and burden of CRDs in the future [18–20]. However, our results represent the CRDs burden before COVID-19 pandemic and the impact of pandemic needs to be investigated further [21, 22]. COVID-19 may increase the number of mortality cases due to CRDs due to misdiagnosis of respiratory conditions such as COPD exacerbation during pandemic and also severity of COVID-19 in patients with CRDs [19, 23].

In our study the incidence and prevalence of CRDs were relatively high in young children (under 14 years). Asthma is the most common NCD in children and the prevalence of childhood asthma is high in low- and middle-income countries with an increasing pattern [24]. In utero and parental smoking is an important risk factor for asthma development in children and the high prevalence of CRDs in young adults could be linked to the high smoking prevalence in NAME countries [25, 26]. While COPD is considered as an adult disease, recent evidences suggest that its origin may be in childhood and neonatal respiratory infections, low birth weight, preterm birth and exposure to smoking may contribute to COPD and long-term reduction of lung function in children [27].

Based on our estimations in NAME the ASIR of pneumoconiosis was higher in women during the study period and showed a significant increase after 2000, while ASDR and age-standardized DALYs were higher in men with an overall decreasing pattern compared to women. The decreasing pattern of ASDR in men could be due to improvement in quality of work places [28]; however, the pattern was increasing in women possibly because more women are employed during past decades due to urbanization in the region [29]. Moreover, it had been shown that in NAME, the contribution of silicosis had decreased but the contribution of asbestosis and coal had increase in the measures of pneumoconiosis incident cases [30]. In contrast to our results which showed an increase in number of deaths and DALYs, the number of deaths due to pneumoconiosis decreased annually in U.S.A from 1999 to 2018 [31]. According to the increasing trends of pneumoconiosis in the region, several government-based plans have to take place in order to minimize occupational risk factors, provide healthy and safe environment for all workers, and ban asbestosis.

Smoking is the main risk factor of CRDs and the rate of smoking varies greatly within NAME region and the highest rate of smoking was reported in Jordan, Lebanon, Syria, and Turkey [32]. Smoking rates in women are generally lower than men [33]. For this reason, in the NAME region smoking was not the most prevalent risk factor of CRDs burden in women and household and ambient particulate matter pollution and high BMI were the most prevalent risk factors in women. The rate of smoking among women in Lebanon was reported to be higher compared to women of other countries in the region [34] and this will explain our results that Lebanese woman had the highest CRDs burden attributed to smoking. Water-pipe usage is also increasing in the region and due to misperceptions regarding the safety of water-pipes, the usage is common among women and children [35]. Water-pipes are shown to be associated with several conditions such as cardiovascular disease and pulmonary disease [36].

High BMI is considered as a major modifiable risk factor of asthma in both children and adults [37] and some gender differences have been reported in the association between high BMI and asthma in adolescents [38]. Prevalence of high BMI had increased dramatically in NAME during past decades mainly due to economic transformations, changes in lifestyles and diets and in most countries the change was more prominent in women due to cultural factors and restricted access to sport activities [39].

Ambient particulate matter pollution is another important risk factor for CRDs in the region mainly due to the small size of these particles which are inhaled deeply into lungs. Sources of air pollution in NAME are windblown dust, sea salt, vehicular emissions and traffic-related air pollution, open burning of waste, industrial emissions, low quality fuels, temperature inversion in cold seasons and household emissions [40]. Increasing urbanization over the last decades in the region is another risk factor for increasing incidence of CRDs [41].

Several action plans must be considered in order to reduce the incidence of CRDs in young adults and to prevent the development of lung diseases by reducing exposure to smoking and air pollution, reducing respiratory infections [27] and also reducing the risk of preterm and low-birth weight neonates [42]. Also, policy makers must provide general access to healthcare facilities and medication, as well as trained health professionals [27]. According to the increasing pattern of incidence and prevalence of CRDs in the region, tobacco control and demand reduction frameworks should be implemented in countries, such as increasing tax on tobacco products, banning smoking in public places, educating people about the adverse effects of tobacco especially at young age, educating about the misperceptions regarding water-pipes and banning advertisement of tobacco products [43]. In Saudi Arabia, the modification of tobacco tax policies in 2017, has resulted in significant reduction in cigarette consumption [44]. Moreover, national plans and policies should be developed in order to reduce the burden of high BMI and to address level of activity, effects of urbanization and dietary changes [45]. Several actions will help reducing the burden of air pollution such as providing high quality fuels, providing public and non-motorized transportations, reducing open burning of waste, and providing clean cooking and heating fuels [40].

This study has many strengths such as, systematic data and method usage which could potentially compare burden of CRDs among different countries in NAME between 1990 and 2019, possibly making the results generalizable. Although some limitations are also present. Where data are not available, out-of-sample prediction and imputation methods are employed by GBD and the results depend on the effectiveness of these methods [10]. Another limitation is that GBD 2019 study did not provide data regarding the incidence and prevalence of “other CRDs” and thus, we only reported mortality and DALYs. Also, there is a lack of standardized case definition across countries in order to distinguish different CRDs. Accurate diagnosis of some CRDs requires equipment and resources such as spirometry which may be lacking in less-developed countries [46]. Moreover, in some countries such as Africa, there is lack of functional vital registration systems [47]. Additionally, the issue of confounders especially between air pollution and smoking has not been taken into account [9].

## Conclusion

In NAME, the number of incident cases and deaths due to CRDs were increasing and COPD and asthma were the most common CRDs in the region. Smoking was the leading risk factor for CRDs especially in men. According to the high incidence and prevalence of CRDs in the region and the fact that much of CRDs’ burden is preventable through appropriate interventions and policies, in this study we tried to provide more attentions on CRDs as an important leading cause of death in the region.

## Supplementary Information

**Additional file 1: Table S1.** List of ICD-10 mapped codes for CRDs.

**Additional file 2: Table S2.** SDI Categories for North Africa and Middle East’s countries

**Additional file 3: Table S3.** Age-standardized rate of incidence, prevalence, deaths, DALYs, YLLs, and YLDs due to CRDs in North Africa and Middle East countries between 1990 and 2019, with percentage change by sex.

**Additional file 4: Table S4.** Decomposition analysis of CRDs new cases between 1990 and 2019 by sex, in North Africa and Middle East region and its countries.

**Additional file 5: Figure S1.** Age-standardized death rates due to asthma, pneumoconiosis and COPD attributable to 12 risk factors by sex in 1990 and 2019.

## Data Availability

The datasets generated and/or analyzed during the current study are available in the GBD repository, https://www.healthdata.org/data-visualization/gbd-compare.

## Abbreviations

ASDR: Age-standardized death rate
ASIR: Age-standardized incidence rate
ASPR: Age-standardized prevalence rate
BMI: Body-mass index
COPD: Chronic obstructive pulmonary disease
CRD: Chronic respiratory disease
DALYs: Disability-adjusted life years
GBD: Global Burden of Disease
ILD: Interstitial lung disease
MIR: Mortality-to-incidence ratio
NAME: North Africa and Middle East
NCD: Non-communicable diseases
SDI: Socio-demographic index
UI: Uncertainty interval
WHO: World Health Organization
YLD: Years lived with disability
YLL: Years of life lost
