# Supplementary material for: The burden of chronic respiratory disease and attributable risk factors in North Africa and Middle East: findings from global burden of disease study (GBD) 2019
Source: Respir Res. 2022 Sep 29;23:268. doi: 10.1186/s12931-022-02187-3 (PMC9521864; doi:10.1186/s12931-022-02187-3)
Supplement: Supplementary file 1 — Additional file 1: Table S1. List of ICD-10 mapped codes for CRDs. [file 12931_2022_2187_MOESM1_ESM.pdf]

International Classification of Diseases-10 codes of diseases included in the current study

Disease	ICD-10 code
**Chronic respiratory disease**	D86-D86.2, D86.9, G47.3-G47.39, J30-J35.9, J37-J39.9, J41-J42.4, J43-J46.0, J47-J47.9, J60-J68.9, J70.8-J70.9, J80-J80.9, J82, J84-J84.9, J90-J90.0, J91, J91.8-J93.12, J93.8-J94.9, J96-J96.92, J98-J99.8, R05.0-R06.9, R09-R09.89, R84-R84.9, R91-R91.8, Z82.5
**Chronic obstructive pulmonary disease**	J41-J42.4, J43-J44.9
**Pneumoconiosis**	J60-J65.0, J92.0
**Asthma**	J45-J46.0, Z82.5
**Interstitial lung disease and pulmonary sarcoidosis**	D86-D86.2, D86.9, J84-J84.9
**Other chronic respiratory diseases**	J30-J35.9, J37-J39.9, J47-J47.9, J66-J68.9, J70.8-J70.9, J82, J90-J90.0, J91, J91.8-J92, J92.9-J93.12, J93.8-J94.9, J96.1-J96.8, J98-J99.8
