# Supplementary material for: The burden of chronic respiratory disease and attributable risk factors in North Africa and Middle East: findings from global burden of disease study (GBD) 2019
Source: Respir Res. 2022 Sep 29;23:268. doi: 10.1186/s12931-022-02187-3 (PMC9521864; doi:10.1186/s12931-022-02187-3)
Supplement: Supplementary file 2 — Additional file 2: Table S2. SDI Categories for North Africa and Middle East’s countries [file 12931_2022_2187_MOESM2_ESM.pdf]

SDI Categories for North Africa and Middle East's countries				
Country	Year 1990		Year 2019	
	SDI	SDI Quintiles	SDI	SDI Quintiles
Afghanistan	0.187	Low	0.343	Low
Algeria	0.436	Middle	0.652	Low-Middle
Bahrain	0.553	High	0.751	High-Middle
Egypt	0.403	Low-Middle	0.658	Low-Middle
Iran (Islamic Republic of)	0.404	Low-Middle	0.67	Low-Middle
Iraq	0.392	Low-Middle	0.671	Middle
Jordan	0.52	High-Middle	0.731	High-Middle
Kuwait	0.655	High	0.851	High
Lebanon	0.462	High-Middle	0.708	Middle
Libya	0.405	Middle	0.709	Middle
Morocco	0.347	Low	0.548	Low
Oman	0.441	Middle	0.783	High-Middle
Palestine	0.314	Low	0.588	Low
Qatar	0.585	High	0.83	High
Saudi Arabia	0.48	High-Middle	0.805	High
Sudan	0.227	Low	0.515	Low
Syrian Arab Republic	0.367	Low-Middle	0.619	Low-Middle
Tunisia	0.434	Middle	0.672	Middle
Turkey	0.473	High-Middle	0.748	High-Middle
United Arab Emirates	0.621	High	0.88	High
Yemen	0.176	Low	0.412	Low
