# Supplementary material for: The burden of chronic respiratory disease and attributable risk factors in North Africa and Middle East: findings from global burden of disease study (GBD) 2019
Source: Respir Res. 2022 Sep 29;23:268. doi: 10.1186/s12931-022-02187-3 (PMC9521864; doi:10.1186/s12931-022-02187-3)
Supplement: Supplementary file 3 — Additional file 3: Table S3. Age-standardized rate of incidence, prevalence, deaths, DALYs, YLLs, and YLDs due to CRDs in North Africa and Middle East countries between 1990 and 2019, with percentage change by sex. [file 12931_2022_2187_MOESM3_ESM.pdf]

Country	Measure	Age-standardized rate (per 100,000)						% Change (1990 to 2019)		
		1990			2019					
		Both	Female	Male	Both	Female	Male	Both	Female	Male
Afghanistan	Incidence	972.2 (859 to 1101.9)	1141.4 (1015.4 to 1277.1)	791.5 (683.4 to 917.2)	1019.2 (901.5 to 1166)	1182.1 (1060.6 to 1336.1)	853.1 (733.8 to 992.1)	4.8 (0.3 to 9.1)	3.6 (-2.7 to 9.4)	7.8 (2.5 to 13.3)
	Prevalence	6271.1 (5753.7 to 6836.3)	7855 (7235 to 8501.3)	4570.1 (4086.8 to 5146.6)	6187.2 (5648.4 to 6825.7)	7545.3 (6951.9 to 8210.3)	4804.6 (4265.5 to 5453.7)	-1.3 (-4.9 to 2.3)	-3.9 (-8.1 to 0.4)	5.1 (-0.2 to 10.9)
	Deaths	94.8 (78 to 113)	87.9 (63.7 to 113.7)	100.7 (82 to 120.4)	67.8 (52 to 81.3)	69 (48.3 to 89.2)	66.4 (52 to 79.4)	-28.5 (-46.3 to -11.6)	-21.4 (-44.1 to 4.6)	-34.1 (-50.4 to -15.6)
	DALYs	2417 (2023 to 2857)	2417.9 (1898.3 to 2964)	2383.7 (1936.4 to 2860.6)	1754 (1422.5 to 2077.1)	1899.1 (1469.3 to 2331.8)	1604.5 (1274.4 to 1888.3)	-27.4 (-42.2 to -11.7)	-21.5 (-38.6 to -1.1)	-32.7 (-47.4 to -14.9)
	YLLs	2061 (1666.3 to 2496)	1975.6 (1459.5 to 2526.9)	2118.4 (1669.5 to 2572.4)	1370.9 (1054 to 1682.8)	1432.7 (1033.2 to 1860.4)	1307.6 (999.3 to 1589.8)	-33.5 (-50 to -15.5)	-27.5 (-47.2 to -3.3)	-38.3 (-54.2 to -18.8)
	YLDs	356 (269 to 450.5)	442.3 (332.6 to 564.1)	265.3 (200.7 to 339.2)	383.1 (293.4 to 474.8)	466.5 (356.2 to 584.6)	296.9 (225.9 to 376.2)	7.6 (2.9 to 12.9)	5.5 (0.2 to 11.8)	11.9 (5.4 to 18.8)
Algeria	Incidence	813.3 (710.7 to 937.2)	840.3 (735.1 to 969.6)	784.3 (684.5 to 908.5)	929.1 (799.8 to 1091.6)	957 (829.2 to 1117.5)	901.3 (767.5 to 1068.3)	14.2 (8.4 to 20.7)	13.9 (7.1 to 21.7)	14.9 (7.7 to 24)
	Prevalence	4503.7 (4056.7 to 5081.1)	4583.2 (4109.6 to 5182.6)	4408.1 (3966.4 to 4977.1)	4879.6 (4306.8 to 5592.6)	4870.3 (4278.3 to 5604.4)	4873 (4277.8 to 5591.7)	8.3 (1.7 to 15.2)	6.3 (-1.5 to 14.9)	10.5 (3.1 to 19.3)
	Deaths	53.8 (41.7 to 66.9)	44.5 (26.4 to 61)	61.3 (45.6 to 80.3)	30.4 (24.5 to 37.5)	27.2 (19.9 to 35.9)	33.2 (25.5 to 42.6)	-43.6 (-56 to -27.4)	-38.8 (-54.2 to -1.1)	-45.9 (-60 to -27.2)
	DALYs	1175.9 (960.2 to 1417.9)	1058.7 (723.8 to 1359.1)	1282.1 (1017.6 to 1602.2)	788.6 (668.7 to 929.8)	737.2 (608.9 to 894.9)	836.9 (691.6 to 1019.9)	-32.9 (-45.2 to -18)	-30.4 (-44.7 to -1.7)	-34.7 (-48.5 to -18.1)
	YLLs	906.1 (693.2 to 1149.1)	785.8 (466.4 to 1068.3)	1016.6 (756.3 to 1346.9)	476.1 (380.6 to 591.8)	425.3 (326.6 to 555)	525 (404.7 to 685.6)	-47.5 (-59.7 to -30.9)	-45.9 (-59.9 to -12.7)	-48.4 (-62.1 to -29.9)
	YLDs	269.8 (205.8 to 346.6)	272.9 (206.5 to 351.1)	265.5 (200.4 to 340)	312.5 (239.6 to 396.1)	311.9 (239.4 to 394.7)	311.9 (237.1 to 398.1)	15.8 (9.9 to 22.2)	14.3 (6.5 to 22.2)	17.5 (10.3 to 25.8)

Country	Measure	Age-standardized rate (per 100,000)						% Change (1990 to 2019)		
		1990			2019					
		Both	Female	Male	Both	Female	Male	Both	Female	Male
Bahrain	Incidence	1148.6 (1010.3 to 1308.1)	1234.9 (1092.1 to 1396.4)	1098.9 (960.8 to 1269.1)	1142 (991.4 to 1321.9)	1256.8 (1099.2 to 1445.4)	1084.8 (928.2 to 1263.4)	-0.6 (-5.8 to 4.6)	1.8 (-4.8 to 9)	-1.3 (-7 to 4.8)
	Prevalence	6694.7 (6138.2 to 7383.4)	7360 (6777.9 to 8048.4)	6284.4 (5704.1 to 7024.6)	5462 (4803.1 to 6235.6)	5758 (5151.3 to 6542)	5371.7 (4657.8 to 6215)	-18.4 (-23.3 to -13.4)	-21.8 (-26.5 to -16.5)	-14.5 (-21.1 to -7.8)
	Deaths	78.3 (67.1 to 88.9)	63.9 (48.6 to 76.7)	96.3 (82 to 110.7)	37.2 (30.5 to 45.4)	33.8 (26.8 to 45.2)	41 (32.4 to 50)	-52.6 (-61.7 to -40.1)	-47 (-59.6 to -25)	-57.4 (-67 to -45.8)
	DALYs	1719.6 (1532.5 to 1932.1)	1575.2 (1331 to 1818.6)	1909.7 (1659.7 to 2167.1)	875.8 (751.6 to 1030.6)	876.9 (739.2 to 1079.2)	900.7 (764.3 to 1067.2)	-49.1 (-56.5 to -40.1)	-44.3 (-53.4 to -29.9)	-52.8 (-61 to -43.5)
	YLLs	1297.8 (1124.3 to 1488.9)	1107.5 (876.5 to 1327.6)	1518.2 (1292.7 to 1749.1)	532.2 (431.3 to 662.7)	506.6 (402.1 to 685.9)	568.3 (452.7 to 708.7)	-59 (-67.4 to -47)	-54.3 (-65.2 to -34.3)	-62.6 (-71.2 to -51.4)
	YLDs	421.7 (326.8 to 521.9)	467.6 (362.7 to 574.5)	391.4 (301 to 486.5)	343.6 (264.1 to 431.4)	370.3 (286.4 to 461.6)	332.4 (252 to 423.8)	-18.5 (-22.6 to -14.5)	-20.8 (-25.1 to -16)	-15.1 (-20.8 to -9.3)
Egypt	Incidence	909.2 (787 to 1049.6)	941.7 (812.5 to 1088.4)	878.3 (757.1 to 1019.4)	1042.4 (916.6 to 1193.8)	1065 (927.9 to 1220.4)	1023.4 (896.1 to 1181.5)	14.6 (8.7 to 21)	13.1 (5 to 20.6)	16.5 (10.1 to 23.7)
	Prevalence	5237.3 (4695.1 to 5866.7)	5241.2 (4659 to 5902.8)	5241 (4705.8 to 5872.7)	5983.2 (5399.4 to 6671)	5721.7 (5095.7 to 6389.6)	6164.8 (5574.5 to 6863.1)	14.2 (8.4 to 19.8)	9.2 (1.6 to 16.4)	17.6 (11 to 24.5)
	Deaths	58.8 (53.4 to 68.3)	53 (45.3 to 67.2)	64.6 (57.9 to 72.3)	43.6 (32.3 to 55.4)	42.5 (29.3 to 62)	46 (30.7 to 62.3)	-25.9 (-44.2 to -4.9)	-19.9 (-44.6 to 21.2)	-28.8 (-52.1 to -4.5)
	DALYs	1589.9 (1440.7 to 1751.9)	1450.9 (1257.3 to 1651)	1728.6 (1555 to 1917.6)	1270.7 (1014.4 to 1541.9)	1154.7 (898.1 to 1541.3)	1380.3 (1058.1 to 1719.1)	-20.1 (-34.4 to -3.9)	-20.4 (-37.1 to 4.3)	-20.2 (-37.9 to -0.1)
	YLLs	1276.2 (1147.2 to 1405.8)	1142.8 (964.6 to 1333.2)	1408.6 (1255.9 to 1571.5)	858.5 (627.9 to 1115.9)	766.2 (526 to 1137.5)	952.2 (656.8 to 1277.4)	-32.7 (-50 to -12.6)	-33 (-54 to -1.4)	-32.4 (-53.8 to -8.6)
	YLDs	313.7 (237.8 to 400.2)	308.1 (234.6 to 394.1)	320 (242.5 to 407.9)	412.2 (317.4 to 508)	388.5 (298.1 to 482.6)	428 (329.7 to 534.1)	31.4 (23.4 to 39.7)	26.1 (17 to 36.2)	33.8 (25.1 to 43.7)

Country	Measure	Age-standardized rate (per 100,000)						% Change (1990 to 2019)		
		1990			2019					
		Both	Female	Male	Both	Female	Male	Both	Female	Male
Iran (Islamic Republic of)	Incidence	948.8 (820.2 to 1108.5)	924.3 (805 to 1073.9)	972.4 (829.6 to 1133.8)	932.1 (799.7 to 1091.5)	915.4 (797.9 to 1063.5)	948 (805 to 1112.1)	-1.8 (-4.8 to 1.1)	-1 (-4 to 2)	-2.5 (-5.9 to 0.8)
	Prevalence	5670.3 (5033.6 to 6394.6)	5519.2 (4881.7 to 6239.1)	5818.2 (5150.7 to 6548.9)	5155.4 (4567.2 to 5859.6)	4908.4 (4353.4 to 5582.1)	5400.5 (4774.5 to 6134)	-9.1 (-12.1 to -6.1)	-11.1 (-14.4 to -8.1)	-7.2 (-10.5 to -3.7)
	Deaths	42.6 (36.1 to 52.6)	36.2 (27.2 to 47.8)	49.4 (42.9 to 62.7)	26.9 (23.2 to 29.1)	22.4 (17.5 to 26.1)	31.4 (27.6 to 34)	-36.8 (-49 to -26.6)	-38.1 (-55.1 to -10.1)	-36.4 (-50.7 to -27.3)
	DALYs	1113.2 (983.7 to 1275.4)	979.1 (792.3 to 1180.9)	1241.6 (1100.8 to 1472.1)	794.1 (705.2 to 886.5)	690.4 (600.6 to 784.4)	898.6 (801.8 to 997.7)	-28.7 (-38.1 to -21.2)	-29.5 (-40.4 to -9.6)	-27.6 (-39.9 to -20.1)
	YLLs	786.2 (687.2 to 946.8)	655.8 (496 to 828.4)	911.4 (804.5 to 1136.2)	456 (405.2 to 492.8)	362.5 (295.3 to 426.8)	550.1 (492.9 to 589.9)	-42 (-52.6 to -32.9)	-44.7 (-56.8 to -17)	-39.6 (-52.9 to -30.5)
	YLDs	327 (247.1 to 419.3)	323.3 (248.1 to 410.7)	330.3 (246.9 to 424.5)	338.1 (262.7 to 420.2)	327.9 (255.1 to 408.5)	348.5 (267.8 to 437.6)	3.4 (-1.4 to 8.7)	1.4 (-3.7 to 6.7)	5.5 (0.3 to 12)
Iraq	Incidence	993.3 (859 to 1146.4)	942 (820.2 to 1087.8)	1045.3 (899.5 to 1220.5)	886.3 (754.2 to 1055.9)	925.3 (794.5 to 1093)	847 (709.6 to 1017.7)	-10.8 (-16.5 to -3.8)	-1.8 (-8.5 to 5.7)	-19 (-25.2 to -11.7)
	Prevalence	6283 (5687.3 to 7012.1)	5794.1 (5222 to 6444)	6786.8 (6188.4 to 7586.4)	4426.3 (3873.2 to 5168.3)	4625.9 (4058 to 5353.8)	4233.3 (3655.4 to 5028.4)	-29.6 (-34.7 to -23.9)	-20.2 (-26.3 to -13.9)	-37.6 (-43.3 to -31.7)
	Deaths	28.2 (22.8 to 35.3)	19.5 (13.6 to 28.7)	38 (29.7 to 47.2)	17.3 (13.8 to 22)	12.6 (9.7 to 18.6)	22.9 (18.2 to 27.9)	-38.5 (-53.8 to -19.5)	-35.2 (-54.9 to -2.9)	-39.8 (-56.9 to -17.7)
	DALYs	947 (804.1 to 1126.3)	769.9 (602.4 to 952)	1138.1 (931.2 to 1374.6)	585.9 (485.3 to 699.7)	529.5 (433.8 to 644.8)	652.2 (533.5 to 777.1)	-38.1 (-49.4 to -25.8)	-31.2 (-44.9 to -12.7)	-42.7 (-54.7 to -28.2)
	YLLs	605.8 (489.2 to 749.1)	445.1 (306 to 604.3)	779 (599 to 987.7)	335.4 (260.6 to 422.2)	259.5 (195.2 to 349.5)	420.6 (327.5 to 518.2)	-44.6 (-59.4 to -25.6)	-41.7 (-60.8 to -7.8)	-46 (-62.8 to -23.6)
	YLDs	341.2 (252 to 439.2)	324.7 (240.8 to 418.5)	359.1 (261.6 to 464.9)	250.5 (186.4 to 329)	270.1 (202.3 to 351.2)	231.5 (169.7 to 311.5)	-26.6 (-31.2 to -21.6)	-16.8 (-22.8 to -10.6)	-35.5 (-40.8 to -30)

Country	Measure	Age-standardized rate (per 100,000)						% Change (1990 to 2019)		
		1990			2019					
		Both	Female	Male	Both	Female	Male	Both	Female	Male
Jordan	Incidence	1097.6 (943.9 to 1275.2)	1127.9 (968.2 to 1310.9)	1072.5 (920.7 to 1250.6)	1178.3 (1012.1 to 1369.8)	1192.3 (1018.1 to 1382.2)	1171.3 (989.6 to 1385.8)	7.4 (2 to 14)	5.7 (-1.5 to 13.8)	9.2 (2.6 to 16.7)
	Prevalence	5861.2 (5170.9 to 6691.6)	5609.6 (4927.5 to 6410.7)	6113.7 (5345.7 to 6991.9)	5524 (4789 to 6462.4)	5236 (4513.7 to 6179.7)	5803.4 (5016.2 to 6764.2)	-5.8 (-11.5 to 1.9)	-6.7 (-13.9 to 2.4)	-5.1 (-11.6 to 3.6)
	Deaths	38.8 (31.7 to 46.2)	33.2 (22.6 to 41.7)	44.6 (35.6 to 55.1)	17.8 (14.6 to 21.5)	13.1 (10.3 to 17.2)	22 (17 to 27.9)	-54.1 (-64.9 to -40)	-60.6 (-71.6 to -35.9)	-50.7 (-65.9 to -31.4)
	DALYs	1051.1 (893.4 to 1214.8)	943.5 (724.5 to 1119.1)	1162.8 (967 to 1367.5)	632.4 (530.8 to 747.8)	530.1 (435.4 to 643.1)	726.9 (597.1 to 871.7)	-39.8 (-49.3 to -29.4)	-43.8 (-54.6 to -25.5)	-37.5 (-50.6 to -23.2)
	YLLs	707.2 (577.7 to 837)	614.8 (405.6 to 762.4)	803.3 (637 to 1002.9)	303.9 (249.5 to 363.8)	221.3 (172.9 to 289.1)	379.6 (295.5 to 478.8)	-57 (-67.3 to -44)	-64 (-74.3 to -40.8)	-52.7 (-67.6 to -34.7)
	YLDs	343.9 (258.7 to 446.1)	328.7 (247.7 to 425.1)	359.5 (267.9 to 466.3)	328.5 (246.8 to 425)	308.7 (231.5 to 401.9)	347.3 (259.1 to 449.2)	-4.5 (-9.6 to 1.7)	-6.1 (-12.6 to 1.5)	-3.4 (-9.6 to 3.9)
Kuwait	Incidence	1020.3 (872.4 to 1186.9)	1076.3 (930.4 to 1244.5)	1006.9 (838.4 to 1200.2)	1089.6 (915.6 to 1291.1)	1154.2 (963.2 to 1365.4)	1050 (875.7 to 1270.4)	6.8 (-0.4 to 14.5)	7.2 (-2.1 to 17.2)	4.3 (-3.3 to 13.3)
	Prevalence	5355.5 (4646.9 to 6201.1)	5216.7 (4571.3 to 6004.9)	5587.4 (4761 to 6564.5)	5116.3 (4279 to 6137.9)	4930.6 (4036.5 to 6020.8)	5276.7 (4410.7 to 6347)	-4.5 (-13.8 to 6)	-5.5 (-17.1 to 8.9)	-5.6 (-15 to 5)
	Deaths	17.2 (14.4 to 19.6)	15.8 (12 to 19)	19.1 (15.9 to 22.1)	12.2 (9.7 to 14.9)	8.4 (6.2 to 11.3)	14.8 (11.2 to 18.7)	-29 (-43 to -11.9)	-47 (-61.3 to -23.3)	-22.7 (-40.2 to -0.4)
	DALYs	595.5 (501.7 to 697.7)	589.8 (487.5 to 697.5)	615.8 (515.5 to 731.4)	470.2 (383.1 to 575.6)	412.6 (328.8 to 515.2)	507.9 (407.8 to 622.2)	-21 (-29.9 to -10.9)	-30.1 (-40.6 to -15.8)	-17.5 (-28.5 to -5.2)
	YLLs	315.8 (271.3 to 356.9)	308.4 (236.4 to 365.6)	330.6 (278.8 to 381.8)	191.9 (154.5 to 233)	138.6 (106.2 to 191.3)	226.6 (174 to 288.9)	-39.2 (-51.3 to -23.7)	-55.1 (-67.2 to -33.8)	-31.5 (-47.7 to -11.1)
	YLDs	279.7 (202.5 to 375.9)	281.4 (206 to 374.9)	285.2 (203.5 to 385.5)	278.3 (201.4 to 376.1)	274 (198.1 to 369.8)	281.4 (201.3 to 379.3)	-0.5 (-8.7 to 7.9)	-2.6 (-12.4 to 8.6)	-1.4 (-10.2 to 8.5)

Country	Measure	Age-standardized rate (per 100,000)						% Change (1990 to 2019)		
		1990			2019					
		Both	Female	Male	Both	Female	Male	Both	Female	Male
Lebanon	Incidence	1008.1 (871.8 to 1178.4)	1023.8 (890.9 to 1196.4)	990.3 (846.7 to 1166.7)	1165.2 (1008.8 to 1352.9)	1190 (1038.5 to 1373.1)	1134.3 (975.1 to 1331.1)	15.6 (9.7 to 21.1)	16.2 (9.1 to 23.4)	14.5 (7.8 to 21.5)
	Prevalence	6195.7 (5550.1 to 6968.9)	6199 (5509.8 to 6989.3)	6179.9 (5494.3 to 6992.3)	6840.3 (6054.3 to 7766.5)	6813.7 (6004.3 to 7780.3)	6859.9 (6063.6 to 7795.4)	10.4 (4.3 to 16.8)	9.9 (2.2 to 17.7)	11 (4.7 to 18.7)
	Deaths	37.8 (30.6 to 47.4)	31.6 (23.1 to 41.9)	44.9 (34 to 60.8)	23.3 (18.1 to 29.7)	16.9 (11.5 to 25.7)	31.5 (21 to 39.5)	-38.3 (-53.4 to -23.9)	-46.6 (-62 to -2.8)	-29.8 (-54.9 to -6.9)
	DALYs	1053 (886.8 to 1241.3)	942.3 (755.9 to 1135.9)	1169.8 (950.7 to 1447.9)	850.4 (708.1 to 992.9)	733 (596.6 to 902.5)	993.8 (797.1 to 1181.7)	-19.2 (-32.4 to -7.3)	-22.2 (-35.7 to 1.3)	-15 (-35.4 to 2.1)
	YLLs	681.7 (546.2 to 848.5)	575.8 (423.4 to 757)	793.3 (599.1 to 1066.3)	399.3 (305.8 to 502.6)	287 (197.6 to 425.4)	536.6 (365 to 684.4)	-41.4 (-56.8 to -26.3)	-50.2 (-64.5 to -14.9)	-32.4 (-55.9 to -9.6)
	YLDs	371.3 (280.3 to 473.5)	366.5 (275.6 to 467.2)	376.4 (282.7 to 481.7)	451 (347.4 to 559.6)	446 (341 to 555.3)	457.2 (349.6 to 569)	21.5 (13.6 to 30)	21.7 (11.8 to 33.4)	21.4 (12.5 to 31.7)
Libya	Incidence	967.7 (845 to 1116.1)	981.1 (857.7 to 1127.5)	963.6 (834.4 to 1116.6)	1032.1 (904.7 to 1189.4)	1038.6 (909.5 to 1200.1)	1026.3 (886.8 to 1187)	6.6 (2.6 to 10.8)	5.9 (-0.1 to 11.5)	6.5 (1.4 to 12.5)
	Prevalence	5780.9 (5226.5 to 6492.4)	5715.1 (5180.3 to 6394.1)	5886.9 (5302.9 to 6620.8)	5961.9 (5335.9 to 6756.3)	5744.4 (5154.5 to 6515.4)	6179.9 (5488.8 to 7024.9)	3.1 (-1.2 to 7.2)	0.5 (-4.5 to 6.3)	5 (-0.2 to 10.2)
	Deaths	33.3 (25.3 to 43.5)	26.7 (18.7 to 36.1)	39.8 (29.9 to 57.6)	26.6 (19.9 to 33.3)	20.6 (15.2 to 27.2)	32.6 (23.6 to 43)	-20.2 (-42.8 to 6.2)	-22.9 (-48.5 to 15)	-18.2 (-46.7 to 13.7)
	DALYs	967.4 (794.6 to 1158)	860 (669.6 to 1052)	1072.7 (864.1 to 1360.2)	882.1 (730.7 to 1036.1)	761.2 (622.3 to 917.3)	999.9 (795.2 to 1209.2)	-8.8 (-25.8 to 10.1)	-11.5 (-30.1 to 12.7)	-6.8 (-29.8 to 17.3)
	YLLs	631.2 (488.5 to 806)	529.9 (365.4 to 700.3)	728.5 (550.7 to 1003.2)	488.8 (359.2 to 619)	385.3 (267.3 to 520)	589.1 (417 to 785.9)	-22.6 (-45.3 to 5.7)	-27.3 (-53 to 11.2)	-19.1 (-48.9 to 15.5)
	YLDs	336.2 (253.4 to 428.4)	330.2 (249.8 to 421.8)	344.2 (258.9 to 440.5)	393.3 (302.6 to 488.8)	375.9 (289.8 to 466.5)	410.8 (316.4 to 513.9)	17 (10.6 to 24.4)	13.9 (6.9 to 22)	19.3 (11.3 to 28.2)

Country	Measure	Age-standardized rate (per 100,000)						% Change (1990 to 2019)		
		1990			2019					
		Both	Female	Male	Both	Female	Male	Both	Female	Male
Morocco	Incidence	738.3 (644.7 to 842.5)	755.6 (656.1 to 863.2)	720.7 (626.3 to 824.8)	876.4 (764.2 to 1016.5)	877.2 (768.6 to 1009.5)	876.3 (759.4 to 1021.2)	18.7 (13.2 to 24.4)	16.1 (9.9 to 23)	21.6 (14.9 to 28.1)
	Prevalence	3960.6 (3582.3 to 4402.2)	4039.9 (3636 to 4496)	3882.2 (3484.3 to 4328.2)	4685.5 (4213.6 to 5282.1)	4551.8 (4074.6 to 5117.7)	4826.3 (4337.1 to 5465.7)	18.3 (12.7 to 23.6)	12.7 (6.1 to 19.5)	24.3 (17.3 to 31.8)
	Deaths	43.7 (34.6 to 59.4)	37.3 (19.2 to 50.9)	51.3 (38.7 to 80.5)	37.3 (29.1 to 46.8)	29.5 (20.4 to 39.9)	45.5 (33.7 to 59.8)	-14.7 (-31.8 to 3)	-20.9 (-37.6 to 15.5)	-11.2 (-35 to 15.3)
	DALYs	1082.3 (904.6 to 1338)	996.7 (604 to 1262.6)	1175.4 (939.5 to 1619.8)	965.1 (805.9 to 1141.4)	834.2 (650.9 to 1032)	1102.8 (887.5 to 1334.7)	-10.8 (-25.7 to 3.9)	-16.3 (-31.5 to 13.5)	-6.2 (-25.7 to 15.4)
	YLLs	838.6 (666.2 to 1084.6)	745.6 (347.2 to 1011.1)	939 (718.8 to 1385)	654.9 (501.8 to 817.2)	535.2 (368.3 to 716.6)	780.5 (569.7 to 1006.3)	-21.9 (-39.8 to -4.1)	-28.2 (-45 to 8.8)	-16.9 (-39 to 9)
	YLDs	243.7 (185.6 to 304.7)	251.2 (191 to 316.4)	236.4 (180.9 to 297.5)	310.2 (240.3 to 387.4)	299 (228.2 to 374.1)	322.3 (250.6 to 404.8)	27.3 (21.3 to 33.7)	19 (12.4 to 26.3)	36.3 (27 to 45.9)
Oman	Incidence	753.2 (639.1 to 891.1)	765.4 (651.1 to 902.7)	762.9 (642.5 to 913.6)	1003.6 (852.5 to 1190.9)	1030.7 (881.4 to 1208)	1006.7 (847.9 to 1198.1)	33.2 (25.3 to 45.5)	34.7 (25.3 to 46.8)	32 (22.4 to 44.7)
	Prevalence	3898.1 (3330.4 to 4632.6)	3933.7 (3307.7 to 4689.7)	4010.6 (3413.9 to 4820.9)	5206.5 (4447.5 to 6159.6)	5298.7 (4498.8 to 6339.5)	5317.3 (4547 to 6272.5)	33.6 (23.8 to 47.7)	34.7 (22.9 to 49.1)	32.6 (22.2 to 47.8)
	Deaths	38.5 (28.8 to 51.9)	31.3 (20.2 to 45.8)	49.2 (36 to 74.3)	25.5 (19.5 to 29.6)	20.4 (12.9 to 26.1)	31.5 (23.1 to 37.7)	-33.9 (-53.2 to -10.1)	-34.9 (-60.6 to 6.1)	-36 (-65.7 to -7.4)
	DALYs	913.7 (721.5 to 1173.1)	792.2 (573.8 to 1073.4)	1070.1 (820.5 to 1499.4)	684 (569.7 to 800.3)	625.4 (493.2 to 754.3)	762.1 (623.5 to 895.5)	-25.1 (-41.9 to -5.7)	-21.1 (-43.1 to 9.1)	-28.8 (-52.9 to -4.4)
	YLLs	703.8 (520.4 to 943.1)	582.2 (378.5 to 855.5)	851.6 (614.2 to 1281.4)	392.8 (304.5 to 454.7)	330.4 (217.9 to 415.9)	463.1 (349.9 to 551.9)	-44.2 (-60.2 to -23.6)	-43.2 (-64.7 to -5.5)	-45.6 (-69.5 to -19.9)
	YLDs	209.9 (152.7 to 281)	210 (151.6 to 282.4)	218.4 (159.1 to 292.7)	291.2 (214.2 to 388.4)	295 (215.7 to 392)	299 (221.3 to 399)	38.7 (29.6 to 50.7)	40.5 (29.2 to 53.5)	36.9 (27.3 to 50.6)

Country	Measure	Age-standardized rate (per 100,000)						% Change (1990 to 2019)		
		1990			2019					
		Both	Female	Male	Both	Female	Male	Both	Female	Male
Palestine	Incidence	1101.2 (947.2 to 1267.2)	1143 (978 to 1323.6)	1048.5 (906.4 to 1206)	1175.3 (1006.2 to 1364.8)	1252.1 (1063.5 to 1454.3)	1094.4 (929.2 to 1286)	6.7 (1.5 to 12.7)	9.5 (2.1 to 17.6)	4.4 (-2.1 to 11.5)
	Prevalence	5253.2 (4697.4 to 5939.4)	5061.1 (4504.8 to 5728.8)	5473.4 (4918.4 to 6202.8)	4826.5 (4214.9 to 5597.6)	4815.2 (4194.9 to 5538.8)	4834.5 (4196.2 to 5598)	-8.1 (-13.4 to -2.7)	-4.9 (-10.8 to 1.2)	-11.7 (-18.1 to -5)
	Deaths	44.3 (34.5 to 58.6)	31.8 (20.8 to 43.3)	61 (47.1 to 91.7)	26.9 (22.3 to 31.5)	18.8 (14.9 to 24.2)	39.7 (33.2 to 46.5)	-39.3 (-56.1 to -20.2)	-40.8 (-59.8 to -6.3)	-34.9 (-61.1 to -11.2)
	DALYs	1146.3 (938.8 to 1421.4)	926.9 (689.6 to 1182)	1417.9 (1145.6 to 1927)	768.9 (661.9 to 878)	636.1 (529 to 746.5)	941 (808.1 to 1079.9)	-32.9 (-46.9 to -18.1)	-31.4 (-47.4 to -8.1)	-33.6 (-54.4 to -16.2)
	YLLs	829.7 (637.5 to 1079.3)	626.3 (394.8 to 860.4)	1081.6 (816.7 to 1616.4)	471.5 (392.6 to 549.4)	337.2 (258.5 to 421.8)	645.4 (536.1 to 752.8)	-43.2 (-59.3 to -24.2)	-46.2 (-63.8 to -14.5)	-40.3 (-63.7 to -18.2)
	YLDs	316.6 (241.6 to 405.2)	300.6 (226.1 to 386.2)	336.3 (254.1 to 423.5)	297.4 (225.5 to 382)	298.9 (226.4 to 382.7)	295.6 (224.7 to 380)	-6.1 (-10.9 to -1.2)	-0.6 (-6.8 to 5.8)	-12.1 (-17.6 to -6.1)
Qatar	Incidence	920.4 (783.5 to 1080.6)	1068.4 (924.5 to 1254.7)	857.4 (725.3 to 1013.6)	972.7 (836.3 to 1147.2)	1151.9 (997.6 to 1337.1)	912.8 (775.1 to 1085)	5.7 (1 to 10.5)	7.8 (1.8 to 14.1)	6.5 (0.3 to 12.7)
	Prevalence	5214.1 (4638.8 to 5905.4)	6372.5 (5704.3 to 7225)	4765.3 (4202.9 to 5462.8)	5056.7 (4392.2 to 5876.6)	5995.6 (5215.5 to 6946.6)	4755.8 (4101.9 to 5565.9)	-3 (-8.6 to 2.7)	-5.9 (-12.6 to 0.4)	-0.2 (-7.1 to 7.4)
	Deaths	32.8 (25.4 to 47.5)	31.3 (22.5 to 46.1)	35.3 (26.3 to 55.1)	23 (17.7 to 32.4)	27.9 (21.1 to 40.5)	21.6 (16.3 to 32.2)	-30 (-52 to -5.3)	-11 (-44.6 to 32)	-38.7 (-60.9 to -12)
	DALYs	874.3 (731.7 to 1100.3)	946.1 (763.4 to 1206.5)	877.9 (710.5 to 1177.4)	616.2 (512.3 to 760.8)	734.7 (598.2 to 937.8)	580.9 (478.5 to 727.6)	-29.5 (-43.5 to -15.2)	-22.4 (-40.2 to -1.8)	-33.8 (-50 to -17.5)
	YLLs	549.2 (427.5 to 775.9)	544.9 (395.9 to 794)	580.8 (434 to 891.1)	308.9 (234.9 to 447.2)	373 (279.8 to 551)	291.2 (219.7 to 422)	-43.8 (-60.7 to -22.2)	-31.6 (-55.2 to 4.6)	-49.9 (-67 to -27.5)
	YLDs	325.1 (249.8 to 408.1)	401.2 (307 to 506.6)	297.2 (228.4 to 373.6)	307.3 (232.4 to 392.3)	361.7 (273.3 to 462.1)	289.7 (220.8 to 370.6)	-5.5 (-11.1 to -0.3)	-9.9 (-15.8 to -3.8)	-2.5 (-8.6 to 4.3)

Country	Measure	Age-standardized rate (per 100,000)						% Change (1990 to 2019)		
		1990			2019					
		Both	Female	Male	Both	Female	Male	Both	Female	Male
Saudi Arabia	Incidence	867.1 (755.2 to 984.8)	961.7 (825.1 to 1099.7)	810 (705.2 to 928.2)	1183.2 (1019.6 to 1357.6)	1300.1 (1109.7 to 1504.2)	1121.1 (956.8 to 1309.9)	36.5 (29.7 to 44.6)	35.2 (26.2 to 46)	38.4 (28 to 51.1)
	Prevalence	3444 (3102.3 to 3897.4)	3498.7 (3174.5 to 3875.7)	3459.1 (3068.1 to 3984.9)	4632.7 (4148.4 to 5247.1)	4435.2 (4034.6 to 4911.6)	4839.7 (4222.6 to 5597)	34.5 (27.4 to 43.2)	26.8 (20.6 to 33.8)	39.9 (28.5 to 53.8)
	Deaths	55.4 (41.4 to 75.7)	55.9 (36.9 to 85)	55.3 (40.9 to 89)	31.7 (25.7 to 37.6)	31.1 (22.7 to 40.9)	32 (25.4 to 38.5)	-42.7 (-61.1 to -21.8)	-44.4 (-66.2 to -8.1)	-42.1 (-68.7 to -17.7)
	DALYs	1215.5 (950.9 to 1598.4)	1284.6 (926 to 1838)	1175.9 (908.1 to 1774.7)	864.4 (740.5 to 998.3)	862.9 (706.3 to 1040.3)	867.6 (729.2 to 1014.2)	-28.9 (-48.9 to -8.7)	-32.8 (-54.9 to -2.2)	-26.2 (-54.1 to -1.1)
	YLLs	994.4 (750.2 to 1370.7)	1043.8 (696.6 to 1592.2)	965.8 (709.4 to 1544.5)	550.4 (444.1 to 661.2)	541.3 (408.9 to 713)	554.9 (438.9 to 675.8)	-44.7 (-63.3 to -22.5)	-48.1 (-69.4 to -13.2)	-42.5 (-68.2 to -16.8)
	YLDs	221.2 (170.8 to 275.3)	240.8 (189.6 to 293.1)	210.1 (160.2 to 268.2)	314 (243.8 to 385.3)	321.6 (251.4 to 388.9)	312.6 (239.8 to 391.8)	42 (34.9 to 49.8)	33.6 (25.6 to 41.9)	48.8 (37.4 to 61.3)
Sudan	Incidence	955.8 (814.4 to 1117.9)	990.5 (851.2 to 1151.3)	919.6 (770.2 to 1093.7)	1026.2 (882.3 to 1198.5)	1062.7 (919.5 to 1234.2)	990.9 (839.1 to 1176.4)	7.4 (1.2 to 12.5)	7.3 (-0.4 to 14.4)	7.8 (0.9 to 14.3)
	Prevalence	5721.8 (5089.7 to 6479.4)	5892.9 (5216.1 to 6657.6)	5546.9 (4876.3 to 6347.3)	5845.5 (5174.4 to 6673.4)	5888.5 (5184.3 to 6706.4)	5785.3 (5053.7 to 6662)	2.2 (-3.3 to 8.4)	-0.1 (-6.9 to 7.4)	4.3 (-2.8 to 12)
	Deaths	72.7 (45.5 to 100.4)	56.7 (31.2 to 95.5)	88.5 (48.1 to 128.7)	45.2 (30.7 to 61)	33.7 (21.7 to 53.3)	55 (33.8 to 76.8)	-37.9 (-51.1 to -19.5)	-40.6 (-55 to -18.3)	-37.8 (-55.5 to -14.3)
	DALYs	1866.4 (1321.6 to 2410.6)	1589.6 (1079.2 to 2218.1)	2119.8 (1291.2 to 2892)	1206.9 (905.5 to 1549)	1005.2 (759.9 to 1354.3)	1374.4 (944 to 1820.7)	-35.3 (-46.5 to -19.4)	-36.8 (-49.1 to -19.2)	-35.2 (-49.5 to -12.9)
	YLLs	1539.4 (990.6 to 2067.9)	1248.4 (742.1 to 1867.6)	1807.1 (986.1 to 2566.3)	851.5 (568.2 to 1161.4)	645.5 (426 to 981.6)	1024.7 (612.5 to 1448.4)	-44.7 (-56.7 to -26.7)	-48.3 (-61.5 to -29.4)	-43.3 (-59.2 to -19.3)
	YLDs	327 (244.5 to 423.7)	341.1 (255.8 to 440)	312.7 (233.6 to 406.3)	355.4 (270.5 to 455.5)	359.7 (271.8 to 462)	349.7 (265.7 to 450.3)	8.7 (3.4 to 15)	5.4 (-1.2 to 12.7)	11.8 (4.4 to 19.6)

Country	Measure	Age-standardized rate (per 100,000)						% Change (1990 to 2019)		
		1990			2019					
		Both	Female	Male	Both	Female	Male	Both	Female	Male
Syrian Arab Republic	Incidence	887 (780.3 to 1012.6)	991.2 (867.3 to 1137.3)	789 (694.6 to 907.3)	992.7 (867.9 to 1137.9)	1079.1 (940.1 to 1241.7)	897.1 (784 to 1026.8)	11.9 (7 to 17.4)	8.9 (2.5 to 15.6)	13.7 (7.9 to 20.3)
	Prevalence	4875.2 (4446.7 to 5397.7)	5520.8 (5011.3 to 6144.1)	4259 (3887.5 to 4734.1)	5136.9 (4638.7 to 5744.4)	5505.5 (4913 to 6181.7)	4659.6 (4198.2 to 5181.9)	5.4 (0.7 to 10.3)	-0.3 (-6.1 to 5.6)	9.4 (3.7 to 15.7)
	Deaths	45.7 (36.9 to 59.8)	42.2 (29.7 to 60.7)	48.9 (38.2 to 64.4)	35 (26.6 to 46.9)	33.5 (25.7 to 47.7)	37.5 (28 to 52.3)	-23.4 (-43.5 to 2)	-20.5 (-44.5 to 21)	-23.3 (-48.3 to 6)
	DALYs	1230.6 (1033.4 to 1467.7)	1169.3 (899.9 to 1450.8)	1282.5 (1045.3 to 1557.5)	987.4 (817 to 1210.6)	929 (767.6 to 1144.8)	1041.4 (839.8 to 1304.6)	-19.8 (-36.1 to 0.5)	-20.6 (-37.6 to 10.9)	-18.8 (-38.7 to 5.3)
	YLLs	933.6 (754.3 to 1172.9)	844.6 (592.1 to 1149.7)	1012.3 (793.6 to 1273.3)	653.3 (492.3 to 870.3)	582.6 (442.1 to 788.9)	729 (535 to 979.7)	-30 (-49.2 to -3.3)	-31 (-52.3 to 10.9)	-28 (-50.6 to 1.3)
	YLDs	297 (227.7 to 373.5)	324.7 (243.9 to 413.9)	270.2 (208.2 to 337.7)	334.1 (256 to 417.1)	346.3 (264.6 to 435)	312.4 (241.4 to 385.8)	12.5 (7.4 to 18)	6.7 (0.6 to 12.8)	15.6 (8.9 to 22.5)
Tunisia	Incidence	894.4 (784.8 to 1030.2)	864.2 (758.9 to 997.5)	922.9 (807.6 to 1071.5)	1016.3 (886.1 to 1183.4)	974.4 (845.3 to 1133)	1058 (922.5 to 1231.9)	13.6 (8.8 to 19.5)	12.8 (6.8 to 19.3)	14.6 (7.7 to 22.2)
	Prevalence	4980.9 (4488.3 to 5634.9)	4566 (4106.7 to 5175.9)	5384.7 (4857.6 to 6103.5)	5477.2 (4869.8 to 6227.7)	4863.2 (4274.6 to 5579.6)	6123.6 (5450.3 to 6936)	10 (4.9 to 15.9)	6.5 (-0.2 to 13.7)	13.7 (7.5 to 20.8)
	Deaths	34.3 (27.6 to 45.5)	27.1 (19.2 to 39.9)	42.3 (32.9 to 57.8)	24 (18 to 31.6)	17.6 (12.3 to 25.7)	31.7 (22.6 to 43)	-30 (-48.2 to -7)	-35.1 (-54.5 to -1.8)	-25.2 (-48.7 to 5)
	DALYs	912 (773.7 to 1118.8)	781.2 (619.5 to 1032.8)	1041.3 (864.5 to 1315.1)	755.2 (615.2 to 910.4)	604.4 (486.6 to 748.8)	920.9 (735.4 to 1133.8)	-17.2 (-32.1 to -0.8)	-22.6 (-37.6 to -1.9)	-11.6 (-30.8 to 9.9)
	YLLs	618.4 (503.2 to 813.9)	513 (371.1 to 750.3)	722.6 (568.3 to 980.4)	406.3 (301.1 to 541.3)	301 (213.6 to 440)	522.6 (372.1 to 719.5)	-34.3 (-52.1 to -11)	-41.3 (-58.8 to -12.6)	-27.7 (-50.5 to 3)
	YLDs	293.7 (222.6 to 376.9)	268.2 (203.1 to 343.1)	318.8 (241.9 to 408.2)	349 (269.9 to 439.4)	303.4 (231.8 to 381.3)	398.3 (307.7 to 496.2)	18.8 (13 to 24.8)	13.1 (6.4 to 20.2)	25 (17.4 to 32.9)

Country	Measure	Age-standardized rate (per 100,000)						% Change (1990 to 2019)		
		1990			2019					
		Both	Female	Male	Both	Female	Male	Both	Female	Male
Turkey	Incidence	1148 (1015 to 1309.9)	1250 (1106.1 to 1449.6)	1043.8 (913.1 to 1192.3)	1294.2 (1147.2 to 1466.3)	1341.2 (1187.4 to 1519.9)	1251.6 (1110.2 to 1423.6)	12.7 (7.3 to 18.3)	7.3 (0.4 to 14.1)	19.9 (13.5 to 27.2)
	Prevalence	7652.9 (7011.3 to 8498.5)	8243.1 (7413.5 to 9289.6)	7059.6 (6483.9 to 7800.9)	7856.2 (7167.3 to 8742)	8064.5 (7235.1 to 9070.7)	7714.7 (7071.1 to 8467.6)	2.7 (-2.3 to 7.6)	-2.2 (-8.4 to 4.4)	9.3 (3.7 to 14.7)
	Deaths	60.2 (50.4 to 68.6)	43 (31.1 to 52.5)	80.4 (64.6 to 95.8)	40.6 (28 to 50.3)	28.1 (16.5 to 35.9)	56 (38.9 to 68.9)	-32.4 (-49.9 to -14.9)	-34.6 (-55.8 to -12.3)	-30.3 (-50.1 to -7.6)
	DALYs	1606 (1404 to 1789.8)	1283.6 (1069 to 1495.9)	1971 (1662 to 2277.4)	1171.9 (962.5 to 1368.3)	941.3 (763.9 to 1113.4)	1448.5 (1167.5 to 1694.8)	-27 (-38 to -14.9)	-26.7 (-37.9 to -14.3)	-26.5 (-41.1 to -9.4)
	YLLs	1150.1 (981.3 to 1300)	802.3 (616.9 to 956.1)	1540.2 (1242.7 to 1825.3)	658.9 (468.6 to 811.1)	428.6 (275.1 to 542.1)	928 (648.9 to 1153.2)	-42.7 (-57.4 to -26.9)	-46.6 (-62.2 to -28.6)	-39.7 (-57 to -19)
	YLDs	456 (346.8 to 579.1)	481.3 (365.8 to 610.8)	430.8 (329.5 to 538.1)	513 (399 to 633.5)	512.7 (397.7 to 639.9)	520.5 (404.6 to 637.2)	12.5 (7.3 to 18.4)	6.5 (0.5 to 13.4)	20.8 (13.6 to 28.6)
United Arab Emirates	Incidence	1335.3 (1159.3 to 1531.5)	1394.2 (1194.5 to 1605.9)	1329.8 (1152.2 to 1539.4)	1412.7 (1237.3 to 1622.2)	1556.8 (1363.3 to 1787.8)	1372 (1192.3 to 1590.9)	5.8 (1.3 to 10.6)	11.7 (5.2 to 18.3)	3.2 (-3.6 to 10.2)
	Prevalence	9769.2 (8659.8 to 10878.1)	10043.6 (8578.5 to 11383.3)	9751.8 (8754.1 to 10900.9)	9499.6 (8528.5 to 10588)	10441.4 (9225.3 to 11779.4)	9254.9 (8344.6 to 10337)	-2.8 (-7.3 to 3)	4 (-2.7 to 13.5)	-5.1 (-10.5 to 0.6)
	Deaths	77.6 (61.1 to 104.1)	66.3 (43.8 to 102.9)	83.5 (65.3 to 124.9)	45.5 (35.6 to 58.3)	31.7 (23.4 to 43.5)	50.6 (39 to 68.1)	-41.3 (-57.7 to -20)	-52.2 (-69 to -24)	-39.4 (-59.2 to -17.1)
	DALYs	2129.2 (1751.4 to 2671.4)	1726.2 (1267.8 to 2338.9)	2328.1 (1888.9 to 3076.5)	1523.3 (1249.5 to 1828.4)	1172.5 (948.4 to 1422.6)	1649.3 (1347.8 to 2005.3)	-28.5 (-44 to -9.2)	-32.1 (-50 to -7.8)	-29.2 (-47.8 to -9.4)
	YLLs	1597.9 (1260.8 to 2123.4)	1197.3 (788.1 to 1800.1)	1790.7 (1383.7 to 2515)	959.7 (728.9 to 1207.8)	576 (423.9 to 763)	1094 (826.6 to 1389.3)	-39.9 (-57.7 to -15.7)	-51.9 (-70 to -20.5)	-38.9 (-59.4 to -13.9)
	YLDs	531.4 (393.3 to 695.4)	528.8 (388.5 to 693.4)	537.4 (400.2 to 701.1)	563.6 (424.7 to 717.3)	596.6 (444.3 to 768.9)	555.2 (419.1 to 706.3)	6.1 (0.4 to 12.6)	12.8 (5.9 to 21.9)	3.3 (-3.3 to 10.2)

Country	Measure	Age-standardized rate (per 100,000)						% Change (1990 to 2019)		
		1990			2019					
		Both	Female	Male	Both	Female	Male	Both	Female	Male
Yemen	Incidence	1000.4 (878.3 to 1141.1)	1066.9 (942.5 to 1213.3)	931.9 (812.1 to 1066.8)	995.2 (868.7 to 1152.7)	1017 (889.8 to 1170.4)	972.2 (840.7 to 1128.9)	-0.5 (-4.9 to 4.2)	-4.7 (-10.2 to 0.9)	4.3 (-1.2 to 10.5)
	Prevalence	6307.7 (5780 to 6936.6)	6673.2 (6109.4 to 7322.5)	5935.9 (5396 to 6566.4)	5812.3 (5254.3 to 6464)	5778.5 (5248.9 to 6420.2)	5847.2 (5238.5 to 6559.4)	-7.9 (-11.8 to -3.4)	-13.4 (-18.3 to -8.7)	-1.5 (-6.3 to 3.8)
	Deaths	79.7 (53.6 to 116.7)	67.3 (38.8 to 118)	99.6 (55.9 to 148.2)	52.5 (41.4 to 66.8)	44.2 (31.8 to 59.4)	61.6 (46.1 to 81.4)	-34.1 (-53.9 to -7.9)	-34.3 (-61.8 to 2.3)	-38.2 (-55 to -6.1)
	DALYs	1995.3 (1467.3 to 2689.3)	1786.1 (1202.7 to 2728.8)	2279.1 (1425.7 to 3115.6)	1372.1 (1130.8 to 1678.7)	1231.9 (958.7 to 1541.4)	1520.2 (1210.8 to 1916.9)	-31.2 (-48.6 to -9.7)	-31 (-54.8 to -4.2)	-33.3 (-49.7 to -3.9)
	YLLs	1636.1 (1108.4 to 2335)	1405.4 (834.7 to 2338.8)	1942.1 (1093.4 to 2780)	1006.9 (773.4 to 1309.6)	870.1 (623.7 to 1167.9)	1151.2 (844.2 to 1559.7)	-38.5 (-56.8 to -12.9)	-38.1 (-64 to -4)	-40.7 (-57.8 to -8)
	YLDs	359.2 (269 to 456.5)	380.7 (284.9 to 481.2)	337 (249.6 to 435.8)	365.2 (280 to 459.3)	361.7 (276.8 to 451.8)	369 (282.4 to 464.2)	1.7 (-3.6 to 7.1)	-5 (-10.7 to 1.1)	9.5 (3.1 to 17.3)

Data in parentheses are 95% Uncertainty Intervals (95% UIs)

DALYs=Disability-Adjusted Life Years; YLLs=Years of Life Lost; YLDs=Years Lived with Disability
