# Supplementary material for: The burden of chronic respiratory disease and attributable risk factors in North Africa and Middle East: findings from global burden of disease study (GBD) 2019
Source: Respir Res. 2022 Sep 29;23:268. doi: 10.1186/s12931-022-02187-3 (PMC9521864; doi:10.1186/s12931-022-02187-3)
Supplement: Supplementary file 4 — Additional file 4: Table S4. Decomposition analysis of CRDs new cases between 1990 and 2019 by sex, in North Africa and Middle East region and its countries. [file 12931_2022_2187_MOESM4_ESM.pdf]

Location		Sex	New cases		Expected new cases in 2019		% 1990 - 2019 new cases change cause			% 1990 - 2019 new cases overall change
			1990	2019	Population growth	Population growth + Aging	Population growth	Age structure change	Incidence rate change	
**North Africa and Middle East**		Both	3154020	5803364	5564468	5389298	76.4%	-5.6%	13.1%	84.0%
		Female	1577791	2892959	2742428	2718829	73.8%	-1.5%	11%	83.4%
		Male	1576229	2910406	2820030	2665018	78.9%	-9.8%	15.6%	84.6%
**Country**	Afghanistan	Both	110284	360801	369686	350865	235.2%	-17.1%	9%	227.2%
		Female	62831	195085	202958	194084	223%	-14.1%	1.6%	210.5%
		Male	47453	165716	164978	153486	247.7%	-24.2%	25.8%	249.2%
	Algeria	Both	195686	365668	323897	321096	65.5%	-1.4%	22.8%	86.9%
		Female	99365	185878	164148	164517	65.2%	0.4%	21.5%	87.1%
		Male	96322	179790	159732	156460	65.8%	-3.4%	24.2%	86.7%
	Bahrain	Both	4497	12828	12771	13587	184%	18.2%	-16.9%	185.2%
		Female	2124	5748	5421	5942	155.2%	24.5%	-9.1%	170.6%
		Male	2373	7081	7234	7480	204.8%	10.3%	-16.8%	198.4%
	Egypt	Both	495048	934265	880538	850389	77.9%	-6.1%	16.9%	88.7%
		Female	248909	452342	436720	421175	75.5%	-6.2%	12.5%	81.7%
		Male	246139	481923	443477	428849	80.2%	-5.9%	21.6%	95.8%
	Iran (Islamic Republic of)	Both	545475	731231	785486	754242	44%	-5.7%	-4.2%	34.1%
		Female	255144	355020	369824	366126	44.9%	-1.4%	-4.4%	39.1%
		Male	290332	376211	415449	387904	43.1%	-9.5%	-4%	29.6%
	Iraq	Both	177568	349360	425030	393558	139.4%	-17.7%	-24.9%	96.7%
		Female	81465	177463	194231	184183	138.4%	-12.3%	-8.2%	117.8%
		Male	96103	171897	230900	209365	140.3%	-22.4%	-39%	78.9%
	Jordan	Both	37223	118945	114799	113173	208.4%	-4.4%	15.5%	219.6%
		Female	17620	56749	53142	54211	201.6%	6.1%	14.4%	222.1%
		Male	19602	62196	61672	58782	214.6%	-14.7%	17.4%	217.3%
	Kuwait	Both	15640	39402	39349	37210	151.6%	-13.7%	14%	151.9%
		Female	6793	19159	18584	18154	173.6%	-6.3%	14.8%	182.0%
		Male	8847	20243	20808	19569	135.2%	-14%	7.6%	128.8%
	Lebanon	Both	32832	59704	51899	51566	58.1%	-1%	24.8%	81.8%
		Female	16288	31598	26426	26958	62.2%	3.3%	28.5%	94.0%
		Male	16545	28107	25477	24633	54%	-5.1%	21%	69.9%

Location	Sex	New cases		Expected new cases in 2019		% 1990 - 2019 new cases change cause			% 1990 - 2019 new cases overall change
		1990	2019	Population growth	Population growth + Aging	Population growth	Age structure change	Incidence rate change	
Libya	Both	39110	61083	62174	57962	59%	-10.8%	8%	56.2%
	Female	18842	30409	30352	29116	61.1%	-6.6%	6.9%	61.4%
	Male	20268	30674	31829	29130	57%	-13.3%	7.6%	51.3%
Morocco	Both	177896	296906	252831	251522	42.1%	-0.7%	25.5%	66.9%
	Female	90451	149546	127403	129236	40.9%	2%	22.5%	65.3%
	Male	87445	147360	125398	122152	43.4%	-3.7%	28.8%	68.5%
Oman	Both	14394	36962	33955	29034	135.9%	-34.2%	55.1%	156.8%
	Female	6237	15111	12553	11571	101.3%	-15.7%	56.8%	142.3%
	Male	8157	21851	21260	17347	160.6%	-48%	55.2%	167.9%
Palestine	Both	20094	48648	48114	46146	139.4%	-9.8%	12.5%	142.1%
	Female	10124	24900	23968	23252	136.7%	-7.1%	16.3%	145.9%
	Male	9970	23748	24138	22698	142.1%	-14.4%	10.5%	138.2%
Qatar	Both	3285	19539	21143	18359	543.5%	-84.7%	35.9%	494.7%
	Female	1434	6740	7059	6551	392.2%	-35.5%	13.2%	369.9%
	Male	1851	12799	13305	11522	618.7%	-96.3%	69%	591.4%
Saudi Arabia	Both	109636	303532	244155	225787	122.7%	-16.8%	70.9%	176.9%
	Female	50043	141260	105214	105020	110.2%	-0.4%	72.4%	182.3%
	Male	59593	162272	138565	121241	132.5%	-29.1%	68.9%	172.3%
Sudan	Both	193598	383235	391136	369528	102%	-11.2%	7.1%	98.0%
	Female	93724	188802	188656	181474	101.3%	-7.7%	7.8%	101.4%
	Male	99874	194432	202511	187539	102.8%	-15%	6.9%	94.7%
Syrian Arab Republic	Both	110780	135424	124494	122483	12.4%	-1.8%	11.7%	22.2%
	Female	59106	74566	69750	69223	18%	-0.9%	9%	26.2%
	Male	51674	60858	55293	54141	7%	-2.2%	13%	17.8%
Tunisia	Both	70956	115871	97293	101203	37.1%	5.5%	20.7%	63.3%
	Female	33460	56461	46715	49694	39.6%	8.9%	20.2%	68.7%
	Male	37496	59410	50503	51388	34.7%	2.4%	21.4%	58.4%
Turkey	Both	638625	1031953	869265	902875	36.1%	5.3%	20.2%	61.6%
	Female	344169	547221	470239	499779	36.6%	8.6%	13.8%	59.0%
	Male	294456	484732	399324	402821	35.6%	1.2%	27.8%	64.6%

Location		Sex	New cases		Expected new cases in 2019		% 1990 - 2019 new cases change cause			% 1990 - 2019 new cases overall change
			1990	2019	Population growth	Population growth + Aging	Population growth	Age structure change	Incidence rate change	
	United Arab Emirates	Both	21291	104967	105107	104407	393.7%	-3.3%	2.6%	393.0%
		Female	8013	32734	30684	31593	282.9%	11.3%	14.2%	308.5%
		Male	13279	72233	73570	72698	454.1%	-6.6%	-3.5%	444.0%
	Yemen	Both	137978	287144	316606	295198	129.5%	-15.5%	-5.8%	108.1%
		Female	70587	143228	163232	153651	131.2%	-13.6%	-14.8%	102.9%
		Male	67391	143916	153476	141545	127.7%	-17.7%	3.5%	113.6%
