# Supplementary figures and images for: The burden of chronic respiratory disease and attributable risk factors in North Africa and Middle East: findings from global burden of disease study (GBD) 2019
Source: Respir Res. 2022 Sep 29;23:268. doi: 10.1186/s12931-022-02187-3 (PMC9521864; doi:10.1186/s12931-022-02187-3)

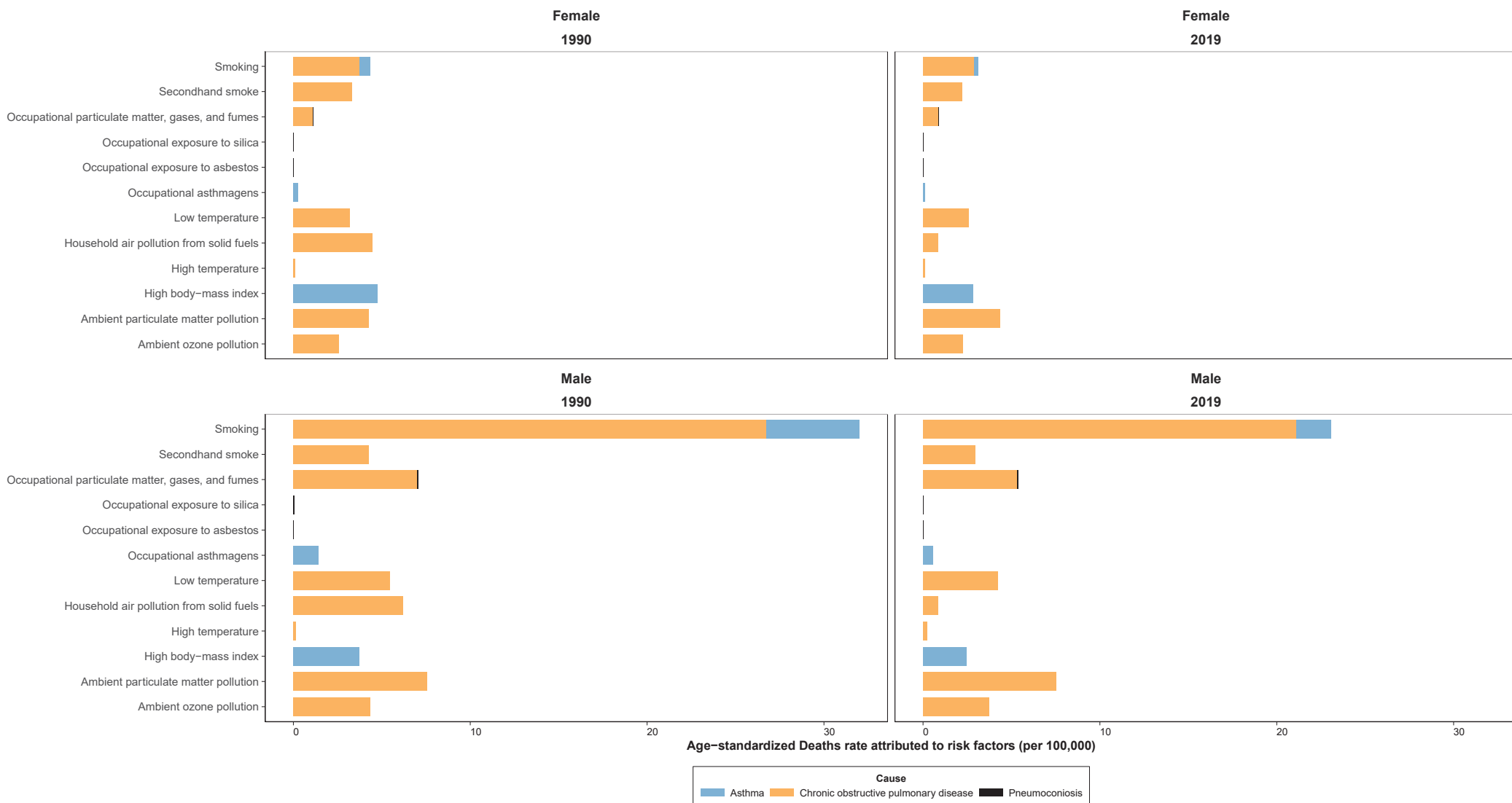

Supplement: Supplementary file 5 — Additional file 5: Figure S1. Age-standardized death rates due to asthma, pneumoconiosis and COPD attributable to 12 risk factors by sex in 1990 and 2019. [file 12931_2022_2187_MOESM5_ESM.pdf]
